# A Novel Computational Strategy to Identify A-to-I RNA Editing Sites by RNA-Seq Data: *De Novo* Detection in Human Spinal Cord Tissue

**DOI:** 10.1371/journal.pone.0044184

**Published:** 2012-09-05

**Authors:** Ernesto Picardi, Angela Gallo, Federica Galeano, Sara Tomaselli, Graziano Pesole

**Affiliations:** 1 Dipartimento di Bioscienze, Biotecnologie e Scienze Farmacologiche, Università di Bari, Bari, Italy; 2 Istituto di Biomembrane e Bioenergetica, Consiglio Nazionale delle Ricerche, Bari, Italy; 3 RNA Editing Laboratory, Oncohaematology Department, Ospedale Pediatrico “Bambino Gesù”, IRCCS, Rome, Italy; Telethon Institute of Genetics and Medicine, Italy

## Abstract

RNA editing is a post-transcriptional process occurring in a wide range of organisms. In human brain, the A-to-I RNA editing, in which individual adenosine (A) bases in pre-mRNA are modified to yield inosine (I), is the most frequent event. Modulating gene expression, RNA editing is essential for cellular homeostasis. Indeed, its deregulation has been linked to several neurological and neurodegenerative diseases. To date, many RNA editing sites have been identified by next generation sequencing technologies employing massive transcriptome sequencing together with whole genome or exome sequencing. While genome and transcriptome reads are not always available for single individuals, RNA-Seq data are widespread through public databases and represent a relevant source of yet unexplored RNA editing sites. In this context, we propose a simple computational strategy to identify genomic positions enriched in novel hypothetical RNA editing events by means of a new two-steps mapping procedure requiring only RNA-Seq data and no a priori knowledge of RNA editing characteristics and genomic reads. We assessed the suitability of our procedure by confirming A-to-I candidates using conventional Sanger sequencing and performing RNA-Seq as well as whole exome sequencing of human spinal cord tissue from a single individual.

## Introduction

RNA editing is a widespread post-transcriptional phenomenon through which primary RNA sequences are altered by nucleotide insertion/deletion or base conversion [1]. RNA editing occurring in mammals, and in particular in humans, involves C-to-U or A-to-I base modifications [1]. While the former are carried out by APOBEC family of deaminases and only few transcripts have been described undergo to this type of editing event [2], the latter, mediated by members of the adenosine deaminase (ADAR) family of enzymes that act on RNA, modify a large number of transcripts [3], [4], including regulatory RNA molecules such as microRNA and their precursors [5]. ADAR enzymes perform the adenosine deamination on double stranded (ds) RNAs through their dsRNA binding domains (dsRDBs) and a conserved C-terminal catalytic domain [4], [6]. In humans, three ADAR genes have been characterized: ADAR1 and ADAR2 encode for active enzymes and are expressed in most tissues and ADAR3, which does not seem to encode for a functional protein and is expressed exclusively in the central nervous system [4].

Inosine is commonly interpreted as guanosine by translation and splicing machineries other than sequencing enzymes. As a consequence, A-to-I modifications can alter codon identity and increase transcriptome as well as proteome diversity [7]. Moreover, RNA editing in pre-mRNAs can generate or destroy splice sites and modify base-pairing interactions within higher-order RNA structures [4], [7].

A peculiarity of RNA editing is that both the edited and unedited versions of affected transcripts are co-expressed in the same cell and the ratio between the two variants can be regulated by a variety of factors depending on tissue type or developmental stage.

A-to-I modifications are prominent in human brain, mostly in untranslated mRNA regions, and indispensable for cellular homeostasis [8], [9], [10]. Indeed, RNA editing deregulation has been linked to several nervous and neurodegenerative diseases such as epilepsy, schizophrenia, major depression and amyotrophic lateral sclerosis [11], [12]. Recent findings also suggest an involvement of RNA editing modifications in human cancer [13], [14], [15], [16].

The most common experimental procedure to discover novel RNA editing events requires the direct comparison between cDNA sequences and the corresponding genomic *locus* of origin in order to look for A-to-G mismatches. Indeed, a large number of A-to-I editing events have been detected by validation (through Sanger sequencing) of A/G mismatches revealed by multiple alignments of mRNA/EST sequences onto the reference genome [17], [18]. However, this approach has numerous limits: 1) it is time-consuming and barely feasible for large-scale RNA/genomic surveys; 2) EST libraries provide only approximate snapshots of the entire human transcriptome and, accordingly, the identification of novel RNA editing sites remains a challenging task; 3) the quality of ESTs is questionable and many alignment mismatches may correspond to sequencing errors rather than genuine RNA editing substitutions.

The advent of high-throughput sequencing technologies have provided unprecedented opportunities for genome-wide investigation of RNA editing aimed at finally obtaining a comprehensive inosinome map [5]. Next generation sequencing shows clear benefits over the classical Sanger methodology, allowing the study of entire genomes and/or transcriptomes and providing deep coverage per reference nucleotide as well as indications of base call qualities. Massive sequencing has been successfully applied to the investigation of C-to-U editing changes in mouse small intestine and plant mitochondrial genomes [2], [19]. In human, the concomitant high-throughput sequencing of genome and transcriptome from the same individual (cell line) has greatly increased the number of known A-to-I editing events, reducing biases due to single nucleotide polymorphisms (SNPs) or somatic mutations [20], [21], [22], [23], [24].

Diverse large-scale RNA editing investigations have been performed up to now showing the complexity of human inosinome, sometimes with questionable results likely depending on different computational strategies of data analysis [20], [21], [22], [23], [24], [25]. This implies that even in the presence of genome and transcriptome data from the same individual, the accurate identification of RNA editing sites is still a challenging task.

Although sequencing costs are rapidly decreasing, the concomitant whole genome and transcriptome sequencing from single individuals and tissues coupled to robust downstream statistical analyses, is still unpractical for large samplings. In contrast, public short read archives contain a huge amount of RNA-Seq data from a variety of human tissues and experimental conditions [26]. Current computational strategies are not yet optimized to detect RNA editing sites from such a huge amount of massive transcriptome data. In addition, the biological role of RNA editing in human gene expression and in several normal as well as pathological conditions is far from being elucidated. We previously developed the ExpEdit web tool to detect known and/or user provided editing events supported by available RNA-Seq data [27]. However, this instrument cannot identify novel but potentially detectable RNA editing events, supported by highly significant and specific A-to-I changes. For this reason, we propose hereafter the first computational methodology to uncover potential A-to-I editing conversions in human mRNAs by massive transcriptome sequencing data without *a priori* knowledge of RNA editing sites or the genomic sequences of the particular donor individual. Starting from a human RNA-Seq experiment our strategy provides a set of genome positions ranked according to their decreasing probability to be edited. We benchmarked this methodology by genuine short sequence reads, focusing on editing conversions in coding protein sequences. Independent Sanger sequencing as well as whole exome and transcriptome reads from human spinal cord of a single individual have been used to experimentally support newly detected A-to-I changes. Our results show that editing events can be reliably predicted *de novo* from RNA-Seq data and highlight that the accuracy of results is strongly biased by the mapping strategy and data quality. We discuss strengths and weaknesses of this approach.

## Materials and Methods

### Data from Public Resources

Illumina paired end RNA-Seq reads (2×50 bp) from SRP002274 study were downloaded from SRA archive using accessions: SRR039628, SRR039629, SRR039630, SRR039631, SRR039632 and SRR039633.

Ensembl (version of 9/Aug/2009), UCSC (version of 10/May/2009) and RefSeq (version of 15/Jul/2011) transcripts for human assembly hg18 were downloaded from UCSC genome browser (http://hgdownload.cse.ucsc.edu/goldenPath/hg18/database/). ASPiCdb transcripts (version of 9/Jun/2009) in GTF format for hg18 human genome were obtained from the following web page: http://www.caspur.it/ASPicDB/.

### RNA-Seq and Exome Mapping

Illumina paired end reads from study SRP002274 and directional reads from spinal cord tissue were aligned by GSNAP (using as main parameters: -B 5 -t 10 -d ann –pairmax-dna = 500 -n 3000–fails-as-input -a paired -O) onto the assembled transcriptome including more than 370,000 transcripts from Ensembl, UCSC, RefSeq and ASPiCdb. Custom python scripts were then used to parse GSNAP results, filtering out inconsistent alignments due to reads with more than two Ns characters and very low quality scores. The same scripts converted transcript coordinates to genome coordinates producing a standard SAM file as output.

Aligned reads were subsequently mapped onto the complete human genome (assembly hg18) by means of GSNAP (using as parameters: -B 5 -d hg18 -t 10 -s splicesites -a paired -O -A sam –no-sam-header) providing a list of exon-exon junctions extracted from the assembled transcriptome and avoiding the prediction of new splice sites. Next, genomic and transcriptomic alignments were compared using a custom python script in order to exclude discordant mappings. Final alignments were printed out in the standard SAM format and converted in the corresponding binary BAM by SAMtools.

Exome reads from spinal cord tissue were aligned onto the complete human genome (version hg18) using GSNAP (with parameters: -d hg18 -B 5 -t 10 -a paired –pairmax-dna = 500 -n 1 -Q -O –nofails –query-unk-mismatch = 1 -A sam –no-sam-header) and requiring only unique mappings. Duplicated reads due to potential PCR artefacts were removed by SAMtools (rmdup program).

### RNA Editing Detection

Read alignments in SAM or BAM formats were converted in pileup format by SAMtools. Such file was parsed by a custom python script and traversed position by position in order to count the number of observed substitutions taking into account a minimum quality score per base of 25. In case of spinal cord RNA-Seq reads we increased the quality score cut-off to 30. The relative frequency of each observed substitution over their global number (versus the reference genome) was used as the empirical distribution of base substitutions for a given dataset (examples of such empirical distributions are shown in Figure S1).

Next for each A-to-G pattern falling in known annotations we calculated a contingency table including the number of observed and expected As and Gs, according to the previously generated empirical distribution of substitutions. The Fisher exact test was applied to evaluate the probability that the observed A-to-G pattern was different from the expected. Significant A-to-G patterns were finally selected at 0.05 confidence level corrected for false discovery rate by Benjamini-Hochberg test [28]. Positions corresponding to known SNPs in dbSNP (version 130) were filtered out before the call of significant sites. The above methodology was implemented in python custom scripts.

### Experimental Validation

Human brain tissues (temporal lobe, cerebellum) and human astrocytoma cell line U118 MG (HTB-15™), or U118 stably transfected with EGFP-ADAR2 cells were used in the study. The study was approved by the local ethic committee. The cells were grown in DMEM supplemented with 10% FCS and antibiotics, at 37°C in 5% CO_2_.

DNA and total RNA were extracted with specific kits (Roche and Invitrogen respectively), according to the manufacturer’s instructions. Each RNA sample was DNase treated (Ambion, Recombinant DNase I) and quantified with the Agilent 2100 bioanalyzer (Agilent). cDNAs were generated by SuperScript II reverse transcriptase (Invitrogen) using random hexamers or specific primers (available upon request). Direct sequencing was performed on DNA and cDNA pools using standard Sanger procedure (Applied Biosystem kit). Direct sequencing was performed on gDNA and cDNA pools, and editing was calculated as described previously [29], [30]. For each sample, 2 independent RT-PCR reactions were performed.

### RNA-Seq, Exome Sequencing and Editing Validation in Spinal Cord

Human spinal cord samples were purchased from the NICHD Brain & Tissue Bank (University of Maryland - http://medschool.umaryland.edu/btbank/). DNA and RNA were isolated, using specific kits (Roche and Invitrogen) after tissue grinding with mortar and pestle. Quantification and integrity were checked by the Agilent 2100 bioanalyzer (Agilent).

Directional RNA-Seq was performed at IGA Institute (http://www.appliedgenomics.org/) using Illumina technology. Libraries were prepared from 2 µg of total RNA adopting a modified protocol based on miRNA sequencing (TruSeq SmallRNA kit). Paired-end reads of 101 bases long were obtained using the Illumina HiSeq2000 machine. Exome sequencing was also performed at IGA Institute from 6 µg of DNA using the Illumina TruSeq Exome Enrichment Kit (62M). Pair-end sequencing was performed on Illumina HiSeq2000.

Significant A-to-G positions in RNA-Seq reads of human spinal cord were detected according to the above described methodology. Such positions were then used to interrogate the corresponding exome BAM file using SAMtools. In case of homozygous positions (at least 5 independent reads with quality score of at least 30) we calculated the statistical support of observing RNA editing using the log-likelihood by perl scripts kindly provided by Iouri Chepelev.

## Results

### Working Hypothesis and Computational Strategy

**Figure 1:**
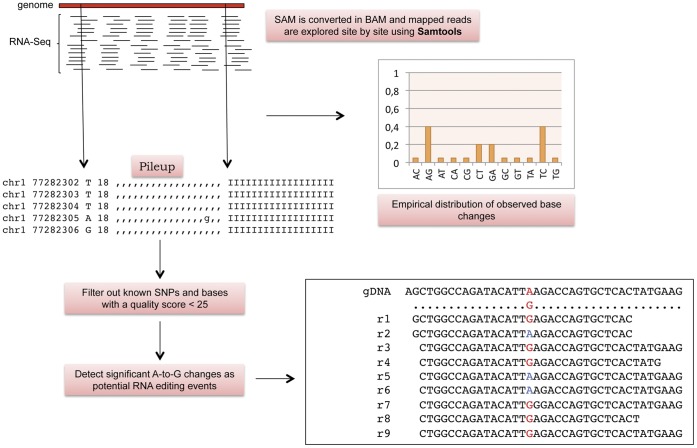
Working hypothesis and computational strategy. Overview of working hypothesis and computational strategy adopted to detect significant A-to-G substitutions in a RNA-Seq experiment.

Potential RNA editing events can be identified looking for A-to-G mismatches in multiple alignments of expressed sequences (ESTs, full length mRNAs or short reads) onto the reference human genome. A-to-G patterns could show different extents ranging from 0 (absence of editing) to 100% (fully edited). This is due to the concomitant presence of edited and unedited transcripts even though some apparent A-to-G substitutions could be caused by SNPs, somatic mutations or sequencing errors. In large-scale experiments involving data from next generation sequencing technologies, we may observe a large number of such A-to-G patterns, many of them being genuine RNA editing conversions. Looking at data from various RNA-Seq experiments, we reasoned that the identification of editing events could be facilitated by considering only those whose occurrence is statistically significant with regard to the empirically observed distribution of base substitutions. For example, given a multiple alignment of short reads onto the reference genome we can calculate empirically the frequency of A-to-G changes and employ such value to verify whether or not the observed A-to-G pattern is significantly different from the expected one by means of the Fisher exact test. Excluding known genomic SNPs and filtering A-to-G patterns by quality scores, we should be able to obtain a set of genomic positions enriched in RNA editing events (Figure 1).

### Mapping Strategy and Editing Identification

A huge amount of deep sequencing data from entire transcriptomes are now freely available through public databases and could be employed to detect *de novo* RNA editing events. To assess the reliability of our working hypothesis and, thus, computational strategy, we downloaded from the SRA database the RNA-Seq experiment SRP002274 containing 23 millions of Illumina paired-end reads 50 bp long, obtained from human brain MAQC mRNA of seven individuals (already used in the past to test algorithms to align spliced reads).

The first challenging task to address for predicting RNA editing events is to correctly align short reads onto the reference human genome reducing biases due to mappings in multiple genome locations or paralogous genes. To date there is no standard procedure to solve this issue and recent works focusing on DNA-RNA differences by deep sequencing take very different approaches and sometimes with questionable findings [20], [21], [22], [23], [25]. Although a variety of mapping programs have been developed, they do not guarantee to find the exact genomic location for each input read and the number of false alignments may dramatically increase depending on user specific parameters and read length/quality.

In first instance we aligned SRP002274 short reads onto the human reference genome by Bowtie program admitting at most two mismatches and discarding mappings in multiple genome locations. Applying our algorithm to discover A-to-I editing events and filtering results for known SNPs, we obtained 55 significant changes in coding regions with 14 of them located in genes, such as PABPC3, GLUD2 or FLJ44635, with recently diverged paralogous counterparts (Table S1). In these cases, the risk exists that such changes could be the result of mapping biases. Therefore, we examined in detail the apparently interesting A-to-G conversion at position chrX:71296770 falling in the coding region of FLJ44635 (NM_207422) gene, highly similar to TPT1 gene on chromosome 13. This A-to-G change was supported by 48 independent and edited reads showing a very low p-value of 7,61×10^−27^ (P<0.05 even when corrected for false discovery rate by Benjamini and Hochberg method [28]). This event would not cause an amino acid replacement. We amplified and sequenced, using the Sanger methodology, genomic DNA and cDNA comprising the candidate site starting from human brain tissue of a single human individual, apparently confirming the predicted RNA editing event (data not shown). However, while alignment of the amplified genomic fragment to the reference human genome recovered the FLJ44635 locus on chromosome X, the optimal alignment of the cDNA sequence was with its paralogous TPT1 locus on chromosome 13. This observation strongly suggests that the hypothetical RNA editing event identified at position chrX:71296770 was a mapping artefact. Indeed, reads supporting the A-to-G change in FLJ44635 gene could be perfectly mapped onto TPT1 mRNA (NM_003295) across an exon-exon junction (data not shown).

To minimize erroneous mappings we aligned again SRP002274 short reads onto the reference genome by Tophat program [31] providing a complete list of known RefSeq exon-exon boundaries. Unique reads with at most two mismatches were maintained for downstream RNA editing prediction in coding regions. The first and last three bases of each read were trimmed to remove spurious substitutions likely due to sequencing errors. The RNA editing analysis yielded 32 significant A-to-G changes, 12 of which were known as edited from literature (Table S2) [8]. However, even this set of predictions did not appear free of erroneous read mappings. Indeed, we found A-to-G changes occurring in the intronless gene GLUD2 on chromosome X, highly similar to GLUD1 on chromosome 10 from which it could have originated by retrotrasposition. Short reads supporting A-to-G changes in GLUD2 could be perfectly mapped onto GLUD1 mRNA (data not shown).

**Figure 2:**
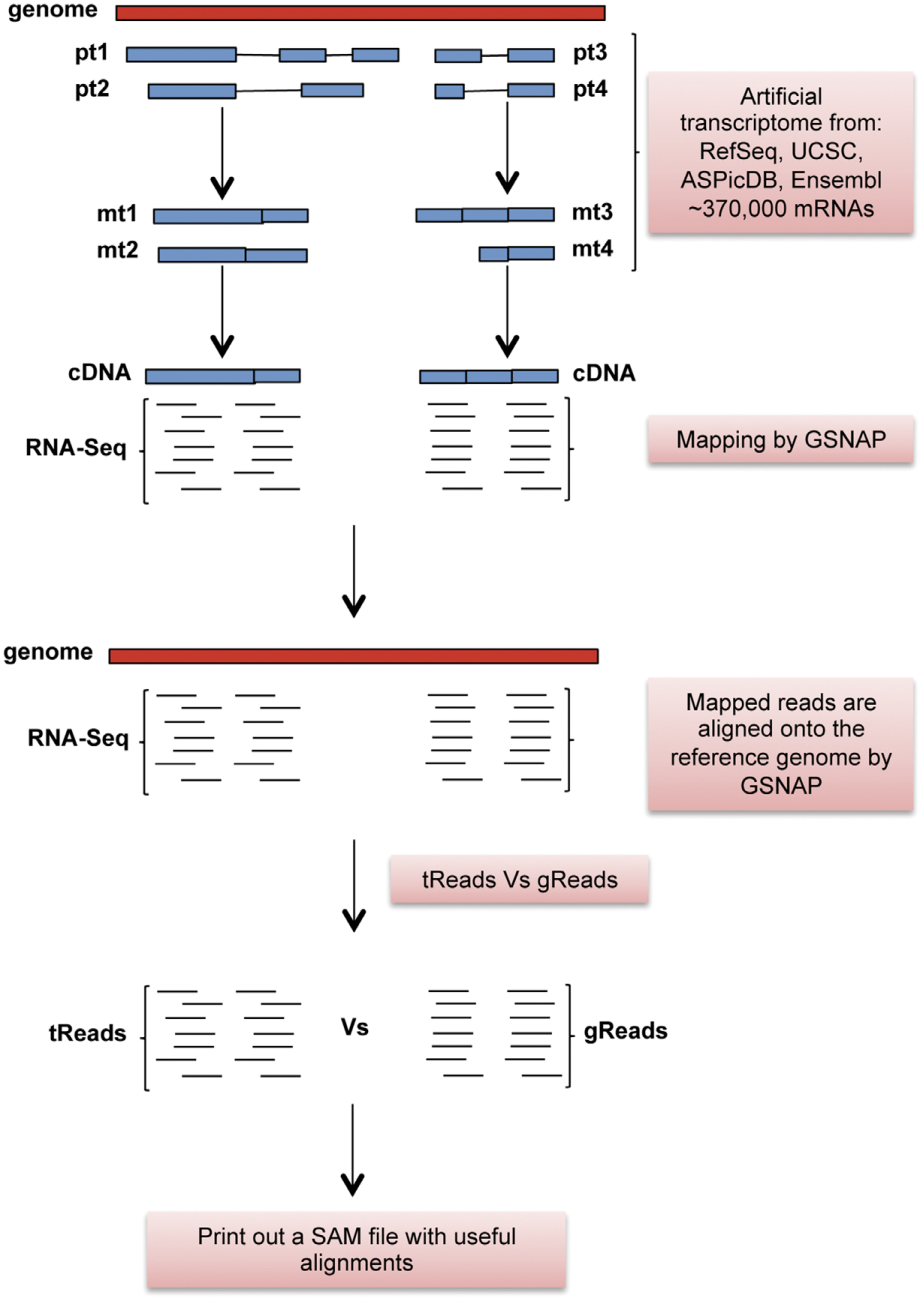
Graphical overview of mapping strategy. Short reads are mapped onto the assembled transcriptome comprising more than 370,000 variants from RefSeq, UCSC, ASPicDB and Ensembl using GSNAP tool. Aligned reads are then mapped onto the complete genome. Finally transcriptome and genome alignments are compared providing a SAM file of unique and concordant mappings.

Accordingly, we further refined our searching strategy to minimize false read alignments and reliably detect A-to-G substitutions linked to RNA editing. To this aim we created a comprehensive human transcriptome collection containing 379,536 transcripts from four widely used databases: RefSeq (36,490) [32], UCSC (50,925) [33], Ensembl (79,931) [34] and ASPicDB (212,190) [35]. SRP002274 short reads were then aligned on this transcriptome data set by the GSNAP program (which enables a flexible tuning of mismatches and auto trimming of low quality alignment regions at read ends [36]). Uniquely mapped reads were subjected to a second alignment round onto the reference human genome, again using GSNAP and providing all exon-exon junctions from the human transcriptome. In the next step, resulting genome alignments were compared with corresponding transcriptome alignments in order to retain only concordant mappings (Figure 2). These reliable multiple alignments of short reads were finally used to calculate the empirical probability of observing base substitutions (focusing on A-to-G changes) excluding known genomic SNPs. A-to-G patterns were subsequently filtered for coding positions according to RefSeq annotations and associated with the probability that each observed pattern was significantly different from that expected (using the Fisher exact test) (Figure S1a). Requiring a base coverage of at least 10 reads, an editing threshold of 10%, and excluding sites with multiple substitutions (other than A-to-G), we recovered 19 RNA editing candidates at a significance level of 0.05 (corrected for false discovery rate by Benjamini and Hochberg method [28]) (Table 1). Interestingly, 11 out of 19 detected positions (58%) were known in published literature and already experimentally validated (Table 1) [8]. Moreover, 17 of the 19 A-to-G changes were recoding and affecting the first (6) and mostly the second (11) codon position in which the Q>R replacement was the most frequent. The estimated RNA editing extent ranged from 10% (as imposed by threshold level) to 100% (49% on average). In addition, the coverage depth per base ranged from 13 to 478 (99 on average) (Table 1).

**Table 1:** List of significant A-to-G changes detected in SRP002274 study.

Position	Gene	Ref	ST	CC	AAC	CodP	CovR	BCR [A, C, G, T]	BCR-F[A, C,G, T]	%Editing	Pvalue	FDR
chr4:57670991	IGFBP7*	T	TC	AAG –> AGG	K –> R	2	263	[0, 167, 0, 96]	[0, 189,0, 173]	63,5	2,62e-66	1,41e-63
chr5:156669386	CYFIP2*	A	AG	AAG –> GAG	K –> E	1	153	[63, 0, 90, 0]	[66, 0,113, 0]	58,82	2,75e-34	7,40e-32
chr1:116739332	ATP1A1	A	AG	ATC –> GTC	I –> V	1	478	[414, 0, 64, 0]	[486, 0,94, 1]	13,39	2,00e-19	3,58e-17
chr4:158500744	GRIA2*	A	AG	AGA –> GGA	R –> G	1	75	[33, 0, 42, 0]	[36, 0,50, 0]	56	2,00e-15	2,69e-13
chr19:59638596	TTYH1	A	AG	CTA –> CTG	L –> L	3	228	[180, 0, 48, 0]	[245, 2,66, 0]	21,05	6,21e-15	6,69e-13
chr1:224041237	SRP9*	A	AG	ATA –> ATG	I –> M	3	57	[26, 0, 31, 0]	[27, 0,41, 0]	54,39	3,47e-11	3,12e-09
chr4:158477325	GRIA2*	A	AG	CAG –> CGG	Q –> R	2	22	[1, 0, 21, 0]	[1, 0,28, 0]	95,45	2,31e-10	1,77e-08
chrX:122426643	GRIA3*	A	AG	AGA –> GGA	R –> G	1	22	[3, 0, 19, 0]	[3, 0,24, 0]	86,36	1,94e-08	1,31e-06
chr15:73433139	NEIL1*	A	AG	AAA –> AGA	K –> R	2	15	[0, 0, 15, 0]	[0, 0,33, 0]	100	1,03e-07	6,18e-06
chr6:44228327	TMEM63B	A	AG	CAG –> CGG	Q –> R	2	92	[70, 0, 22, 0]	[95, 0,24, 0]	23,91	7,73e-07	4,17e-05
chrX:153233144	FLNA*	T	TC	CAG –> CGG	Q –> R	2	67	[0, 20, 0, 47]	[0, 22,0, 50]	29,85	2,30e-06	1,12e-04
chr11:105309904	GRIA4*	A	AG	AGA –> GGA	R –> G	1	19	[4, 0, 15, 0]	[6, 0,17, 0]	78,95	3,35e-06	1,51e-04
chr2:267003	ACP1**	A	AG	CAA –> CGA	Q –> R	2	44	[26, 0, 18, 0]	[26, 0,18, 0]	40,91	5,24e-06	2,17e-04
chr6:102479282	GRIK2*	A	AG	CAG –> CGG	Q –> R	2	13	[2, 0, 11, 0]	[2, 0,11, 0]	84,62	1.06E-04	4,09e-03
chr21:33845189	SON	A	AG	CTA –> CTG	L –> L	3	35	[21, 0, 14, 0]	[26, 0,17, 0]	40	1.17E-04	4,21e-03
chr19:8484035	ZNF414	T	TC	CAG –> CGG	Q –> R	2	54	[0, 13, 0, 41]	[0, 14,0, 55]	24,07	4.51E-04	1,52e-02
chr14:25987370	NOVA1	T	TC	AGC –> GGC	S –> G	1	30	[0, 12, 0, 18]	[0, 14,0, 23]	40	5.25E-04	1,67e-02
chr16:357758	MRPL28**	T	TC	TAT –> TGT	Y –> C	2	104	[0, 13, 0, 91]	[0, 14,0, 104]	12,5	6.50E-04	1,95e-02
chr20:35580977	BLCAP*	T	TC	CAG –> CGG	Q –> R	2	110	[0, 12, 0, 98]	[0, 12,1, 124]	10,91	1.31E-03	3,71e-02

* Positions already known to be edited from literature.

** Positions validated in spinal cord by exome sequencing. Ref: Reference nucleotide; ST: substitution type; CC: codon change; AAC: amino acid change; CodP: codon position; CovR: RNA-Seq coverage; BCR: RNA-Seq base count. i.e. the distribution of supported RNA-Seq bases; BCR-F: RNA-Seq base count before all filtering steps; FDR: false discovery rate.

List of significant A-to-G substitutions in known protein coding regions detected in SRP002274 study and sorted according to ascending Pvalue. For each position corrected Pvalue (FDR), RNA-Seq bases supporting the editing event, codon and aminoacid change (if any), other than gene name, chromosome position and potential editing extent are reported.

An explanatory overview of our computational framework is depicted in Figure 2.

**Figure 3:**
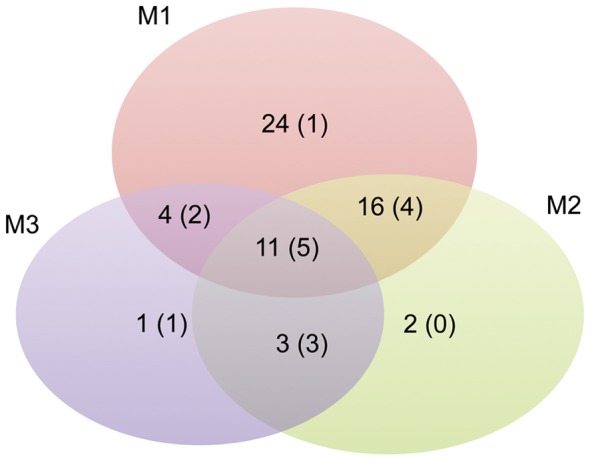
Evaluation of the effectiveness of three different mapping strategies in the editing detection. Venn diagram comparing the effectiveness of three different mapping strategies in the detection of editing sites and reporting overlaps and differences in the number of predicted and literature validated (in brackets) editing events. Mapping strategies are: M1) Bowtie against the reference genome; M2) Tophat against the reference genome; and M3) GSNAP against transcriptome and reference genome.

The better performance of the last strategy with respect to the first and the second one, in the reliable identification of the editing sites, is supported by the observation that the fraction of known editing sites increases from 12/55 (21.8%, Table S1), to 12/32 (37.5%, Table S2), and finally to 11/19 (57.9%, Table 1). A Venn diagram comparing the three different strategies and reporting overlaps and differences in the number of predicted and literature validated editing events is shown in Figure 3. Of course the observed fraction of bona fide editing sites is likely underestimated, as some genuine editing sites may be still not reported in the literature. Although the double mapping strategy reduces the global number of editing candidates, the resulting list is more enriched in genuine RNA editing positions.

### Experimental Validation

**Figure 4:**
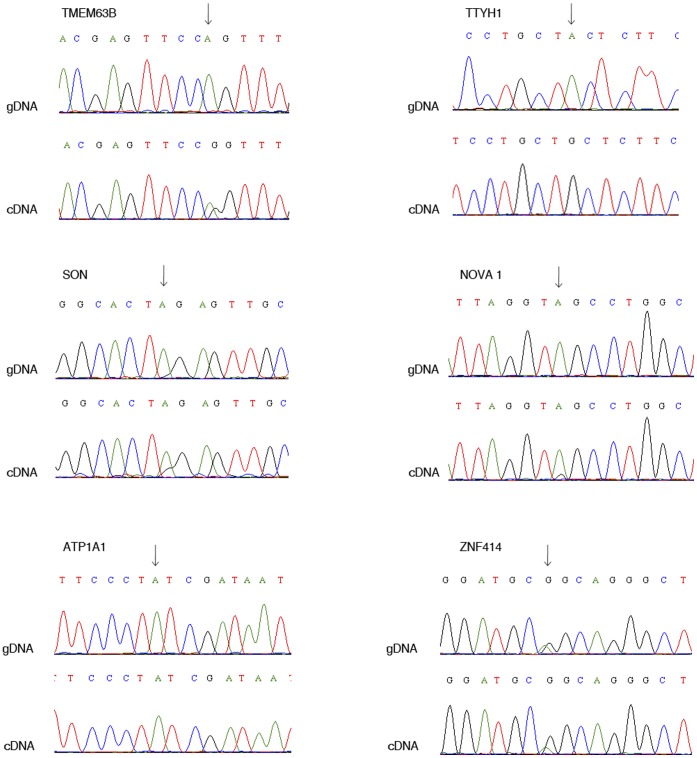
Sanger confirmation of editing candidates. RNA editing events identified within coding regions of candidate genes from SRP002274 study were validated using classical Sanger sequencing method by comparing genomic and the corresponding cDNA portions: a) editing event within the TTYH1 at position chr19:59638596 in human brain; b) editing in TMEM63B at position chr6:44228327 in astrocytoma cell lines over-expressing ADAR2; c) editing in SON at position chr21:33845189, in human brain; d) editing in NOVA1 at position chr14:25987370, in human brain; (e) editing event predicted within ATP1A1 gene did not revealed any editing or SNP when compared both gDNA and cDNA sequences isolated from human brain; (f) we identified a SNP within ZNF414. Chromosome coordinates are referred to human genome assembly hg18.

In order to confirm the effectiveness of our strategy and, thus, demonstrate the specific enrichment of novel RNA editing events, we experimentally assessed the remaining 8/19 predicted editing positions. Two out of these eight editing events, in MRPL28 and ACP1 genes, were experimentally supported by parallel exome and transcriptome sequencing in human spinal cord (see below). The validation of the six remaining sites (in TTYH1, TMEM63B, SON, NOVA1, ATP1A1, ZNF414 genes) was carried out by direct sequencing. In order to exclude potential SNPs at the predicted editing position, we sequenced both the gDNAs and the cDNAs from the same tissues and cell line, i.e. human brain tissue or astrocytoma cell lines. Briefly, total RNA was extracted and RT-PCR was performed using specific primers for the different transcripts, similarly the corresponding DNA portions were amplified and sequenced. The comparison of the gDNA and the cDNA traces revealed that four out of six candidates (TTYH1, TMEM63B, SON, NOVA1) were genuine RNA editing events (Figure 4a–d).

The editing event predicted *in silico* for the ATP1A1 transcript was not supported by Sanger sequencing after comparison of both gDNA and cDNA despite different human sample tissues (temporal lobe and cerebellum) and cell lines were used (Figure 4e and data not shown). However, we cannot exclude the possibility that this editing event may occur in other brain locations/tissues/conditions or that the editing extent is below the detectability level of the sequencing method employed.

Finally, the recoding Q>R (position chr19:8484035) falling in ZNF414 gene was shown to be a genuine SNP (Figure 4f).

In summary, 17 sites out of the 19 predicted candidates in SRP002274 (90% of all predicted sites) resulted real RNA editing events, demonstrating the usefulness of our computational strategy in detecting bona-fide RNA editing sites from RNA-Seq data. This is potentially an underestimate, as invalidated events may occur in other tissues, cell types, physiological or pathological conditions.

### RNA Editing in Human Spinal Cord

A further assessment of our strategy was carried out performing an RNA-Seq experiment on total RNA extracted from human spinal cord. In contrast with the above SRP002274 study, our deep sequencing was accomplished in spinal cord of a single human individual producing paired-end and strand oriented reads using the Illumina platform. Initially, by using GSNAP program, we mapped 40,277,555 RNA-Seq reads onto the human transcriptome and, then, mapped reads (80%) were also aligned to the complete reference genome. Both transcriptome and genome alignments were compared and concordant mappings (73%) were collected for downstream analyses. Multiple alignments of short reads were scanned site by site using SAMtools [37] in order to calculate the probability of observing base substitutions (Figure S1b). Empirical substitution rates were used to evaluate the statistical significance of observed A-to-G changes and focusing on known RefSeq coding protein regions only. At 0.05 significance level, corrected for false discovery rate (by Benjamini and Hochberg method [28]), we obtained 15 RNA editing candidates covered by at least 30 independent reads and showing only A-to-G changes. Among these, three sites, located in SRP9, CCNI and FLNA coding sequences, were experimentally known editing events [8], [38]. In order to validate the remaining 12 editing events we performed a whole exome sequencing by Illumina technology using gDNA sequences isolated from the same spinal cord sample.

After mapping 33,180,939 exome reads onto the reference human genome and excluding potential PCR duplicates, we interrogated the resulting multiple alignments by SAMtools using the list of the 12 RNA editing candidates. Ten out of 12 sites were supported by independent exome reads taking into account only bases with quality scores of at least 30. However, only 6 out of 10 positions were covered by at least 5 reads (quality score ≥30) containing only adenines and thus strongly supporting the presence of editing (Table 2). For such positions we also calculated the likelihood ratio statistic to evaluate the significance of each predicted event, following the methodology proposed by Li et al. [8] and described in detail by Chepelev [39]. This value ranged from 25 to 321, indicating a strong evidence of RNA editing rather than sequencing errors (Table 2).

**Table 2:** Significant A-to-G substitutions detected in RNA-Seq of human spinal cord.

Position	Gene	Ref	ST	CovR	BCR [A, C, G, T]	BCR-F[A, C,G, T]	CovE	BCE [A, C, G, T]	%Editing	Pvalue	LLR
chr9:35688080	TLN1	T	TC	196	[0, 79, 0, 117]	[0, 150,0, 122]	21	[0, 0, 0, 21]	40.31	4.05E-27	321.98
chr9:139026134	ABCA2	T	TC	377	[0, 76, 0, 301]	[0, 152,0, 508]	0	[0, 0, 0, 0]	20.16	8.51E-24	ND
chr16:357758	MRPL28	T	TC	116	[0, 60, 0, 56]	[0, 63,0, 58]	6	[0, 0, 0, 6]	51.72	1.02E-21	176.42
chr1:1299268	AURKAIP1	T	TC	93	[0, 43, 0, 50]	[0, 58,0, 55]	1	[0, 0, 0, 1]	46.24	5.54E-15	120.94
chr17:19624930	ULK2	T	TC	116	[0, 42, 0, 74]	[0, 86,0, 80]	6	[0, 0, 0, 6]	36.21	6.36E-14	111.03
chr4:78198704	CCNI*	T	TC	174	[0, 35, 0, 139]	[0, 54,0, 151]	28	[0, 0, 0, 28]	20.11	8.89E-11	125.07
chr4:191101378	FRG1	A	AG	71	[54, 0, 17, 0]	[56, 0,20, 0]	0	[0, 0, 0, 0]	23.94	2.74E-05	ND
chrX:153233144	FLNA*	T	TC	76	[0, 17, 0, 59]	[0, 51,0, 69]	5	[0, 0, 0, 5]	22.37	2.95E-05	78.04
chr11:61481492	BEST1	A	AG	106	[89, 0, 17, 0]	[95, 0,18, 0]	4	[4, 0, 0, 0]	16.04	3.89E-05	39.64
chr12:6043772	VWF	T	TC	133	[0, 15, 0, 118]	[0, 15,0, 133]	2	[0, 0, 0, 2]	11.28	1.80E-04	29.77
chr4:191115593	FRG1	A	AG	59	[45, 0, 14, 0]	[45, 0,15, 0]	159	[159, 0, 0, 0]	23.73	2.28E-04	35.87
chr17:37235419	NT5C3L	T	TC	45	[0, 13, 0, 32]	[0, 20,1, 67]	2	[0, 0, 0, 2]	28.89	3.82E-04	37.26
chr3:58116831	FLNB	A	AG	105	[92, 0, 13, 0]	[101, 0,15, 0]	11	[11, 0, 0, 0]	12.38	6.52E-04	28.84
chr1:224041237	SRP9*	A	AG	30	[19, 0, 11, 0]	[21, 0,22, 0]	0	[0, 0, 0, 0]	36.67	1.23E-03	ND
chr15:20421437	TUBGCP5	A	AG	53	[42, 0, 11, 0]	[46, 0,16, 0]	8	[8, 0, 0, 0]	20.75	1.96E-03	25.85

* Positions already known to be edited from literature. Ref: Reference nucleotide; ST: substitution type; CovR: RNA-Seq coverage; BCR: RNA-Seq base count. i.e. the distribution of supporting RNA-Seq bases; BCR-F: RNA-Seq base count before all filtering steps; CovE: exome coverage; BCE: exome base count; LLR: log-likelihood ratio.

List of significant A-to-G substitutions in known protein coding regions detected in transcriptome reads from spinal cord. Exome support is also reported for each position as well as Pvalue and log-likelihood ratio [49] [39].

Exome and transcriptome reads from human spinal cord also confirmed editing candidates in MRPL28 and ACP1 genes from SRP002274 study (see previous paragraph). Indeed, both editing events in MRPL28 and ACP1 genes were supported by significant LLR of 176 and 30 respectively, suggesting a high probability of being real RNA editing events.

Although we focused on A-to-I editing in coding protein regions, we used the same computational strategy to predict potential events falling in untranslated regions (UTRs) or alternative exons (AEs) not represented in RefSeq transcripts. In particular, we detected 156 candidates in UTRs (154 in 3′UTR and 2 in 5′UTR) and 21 in AEs (Table S3). Interestingly 120 out of 154 3′UTR A-to-I changes (78%) were located in *Alu* regions and 52 were already annotated in DARNED database as edited sites. In addition, 33 positions were confirmed as genuine RNA editing events by exome reads. Of the 21 sites in AEs, 8 were located in *Alu* elements and 2 confirmed as edited by exome data. Notably, the limited exome data support (35/177 positions) was due to lack of coverage in all cases. Such findings are consistent with the expected distribution of RNA editing events in different mRNA regions, being more frequent in 3′UTRs and *Alu* repeats, than in coding protein sequences or 5′UTRs. However only a small fraction of the detected sites have been validated as genuine RNA editing sites by exome data. This is quite expected, as exome sequencing platforms specifically enrich sequence reads mapping protein-coding exons.

## Discussion

RNA editing is emerging as an important post-transcriptional process increasing the already complex gene expression dynamics in eukaryotic genomes [4], [40]. Editing by base substitution is prominent in plant organelles and especially in mitochondria in which specific cytidines (C) are modified in uridines (U) by deamination. Despite C-to-U conversion being the most frequent, reverse U-to-C changes have also been reported [41], [42]. To date more than 8,000 experimentally verified modifications from plant mitochondria and chloroplasts have been annotated in the specialized REDIdb database [42], [43].

In humans, RNA editing can expand the transcriptome and proteome diversity by the A-to-I and/or C-to-U conversions in primary RNAs. RNA editing changes by adenosine deamination have been investigated in detail during the last two decades using computational methodologies employing ESTs and full-length mRNAs other than intrinsic properties of RNA editing sites as their association to double RNA strands or evolutionary conservation [17], [44]. Such approaches, however, are restricted to specific genomic regions in which dedicated algorithms can find strong statistical evidence of secondary structures and/or conserved sequences in which evolutionary constraints can be assessed. In addition, EST sequences are well known to be error prone and incapable of providing a consistent overview of the transcriptome in a given experimental condition. In a past survey of RNA editing in human brain by Sanger sequencing of a more than 6,700 clones from a cDNA library, no A-to-I event was detected in protein coding exons, despite the fact that almost all currently characterized RNA editing changes in protein coding regions occur in the brain [45]. The advent of next generation sequencing technologies allows the investigation of entire transcriptomes, greatly extending previous studies based on Sanger technology [46], [47]. Some large-scale investigations for RNA editing in humans, employing the huge amount of data from transcriptome and genome, have been proposed [20], [21], [22], [23]. In almost all cases, however, such studies have been conducted on cell lines or human tissues (mostly blood) in which the RNA editing process is not expected to be prominent. Notably, the number of detected DNA-RNA differences was extremely variable and in some cases results have also been questioned [20], [21], [22], [23], [25]. The main problem to address for correctly investigating the impact of RNA editing by massive transcriptome sequencing is to mitigate as much as possible the effect of spurious read alignments. Nowadays, there is not a unique and satisfactory solution to this task even though many mapping tools are available which are fast and memory efficient. In Li at al. [20] paired end Illumina reads from 1000 Genomes Project were aligned onto a transcriptome collection including only Gencode mRNAs. Similarly Ju et al. [21] mapped Illumina paired end reads of eighteen Korean individuals onto an extended transcriptome dataset comprising also UCSC, Ensembl and RefSeq mRNAs. Although the mapping of short reads onto transcript variants has the benefit of accurately detect alignments across exon-exon junctions, it is not exhaustive and misalignments to transcribed paralogous genes could support spurious RNA editing events [25]. Alternatively, in Bahn et al. [22] Illumina RNA-Seq reads were aligned onto the reference human genome using multiple mapping tools adopting different algorithms. Similarly, Peng at al. [23] mapped millions of Illumina transcriptome reads without taking into account exon-exon junctions but including a combination of stringent filters. In contrast li. et al aligned RNA-Seq reads onto human reference genome and a collection of splice site junctions, refining downstream analyses by *ad hoc* filters. Although these strategies overcome limitations of assembled transcriptome datasets, they might not be error free. Indeed, as demonstrated in our work, the mapping of short reads on the complete genome using both tools that do not map across exon-exon boundaries and tools that allow splice alignments, affects the prediction of RNA editing since ambiguous alignments are difficult to be detected and removed. In contrast with current mapping strategies we developed a new framework based on two mapping rounds, the first onto an extended transcriptome collection (including more than 370,000 transcripts) and the second onto the complete genome. In this way read pairs mapping uniquely and consistently on transcripts and genome are maintained for downstream analyses, providing an accurate set of alignments. Minimizing biases due to read mapping, multiple alignments can be interrogated to select patterns of A-to-G changes. Excluding all known SNP positions, we can verify how many observed A-to-G conversions show a pattern significantly different from expected. In this way we greatly reduce the number of A-to-G candidates to investigate for RNA editing. The possibility of recovering reliable candidates is strictly affected by the quality of the original data set, read mapping and the use of filtering procedures to exclude SNPs. Without *a priori* information about known editing events and properties, it is not easy to distinguish genuine RNA editing events from polymorphisms or sequencing errors. However, we currently restrict the selection of significant A-to-G patterns to those in which all supporting bases have a quality score of at least 25 and an editing extent higher than 10%, mitigating potential sequencing errors. According to filtering procedures, therefore, we can provide a ranked list of positions enriched in RNA editing events, addressing experimental validations to a restricted number of candidates. To corroborate our computational strategy we predicted RNA editing candidates in two RNA-Seq experiments with different characteristics. In the SRP002274 study we verified the behaviour of our strategy in a data set enriched in editing events as well as SNPs. Indeed, this RNA-Seq experiment has been performed pooling diverse brain locations from seven different individuals. Results clearly confirm that our method, despite its simplicity, provides a set of reliable RNA editing candidates. Using stringent criteria we identify and confirm six new A-to-I changes in human brain in which four are recoding with potential functional consequences, as for example the RNA editing position at chr14:25987370 in NOVA1 that has been recently associated with protein stability [48]. In the experiment on human spinal cord we have checked the feasibility of our method in finding significant RNA editing events in a tissue not yet well investigated by deep sequencing but in which A-to-I editing could play an important role especially during neurodegeneration. We provide evidence of RNA editing, confirmed by exome sequencing from the same individual, for 8 sites in coding protein genes and 35 sites in UTRs and AEs.

Considering that our procedure requires only RNA-Seq data and, at the time of writing, the SRA database includes more than 4,200 entries of human transcriptomes, it could be used to investigate and discover new RNA editing events, improving our understanding of this basic molecular phenomenon. In addition, RNA-Seq data can now be produced in a timely and cost effective way greatly extending tissue types and experimental conditions to be tested.

Recently a new method to accurately detect RNA editing in the human transcriptome has been reported [22]. Although it is described as a *de novo* RNA-Seq based approach not assuming any prior knowledge about RNA editing process, it requires genomic short reads to exclude genetic polymorphisms and assess the statistical significance of predictions by LLR test. In contrast, our approach is completely *de novo* and does not require *a priori* RNA editing information or genome reads from the same individual. Potential SNPs are filtered out using known annotations in polymorphism databases as dbSNP, making our method useful for any RNA-Seq experiment. In addition, our RNA editing workflow can be easily extended to take into account further filtering criteria – such as the presence of secondary RNA structures or evolutionary conservation. In the presence of genome and/or exome read, the method is still useful to single out genuine editing events after the exclusion of heterozygous genomic sites. Indeed, the p-value calculated by the Fisher exact test performs similarly to LLR test in which very low Fisher p-values correspond to highly significant LLR scores (Figure S2).

In summary, we have developed and tested a new computational approach to predict completely novel RNA editing events from massive transcriptome data sets. Although validated in human samples, it can be applied to provide lists of A-to-I editing candidates in other organisms such as mouse or drosophila.

## Supporting Information

Figure S1 **Examples of empirical distribution of base substitutions.** Empirical distributions of base substitutions for reads from SRA study SRP002274 (a) using a minimum quality score of 25 and from spinal cord RNA-Seq experiment (b) using a minimum quality score of 30.
(TIF)

Figure S2 **LLR score Vs Fisher Pvalue.** Relationship between LLR scores and Fisher Pvalues on 180 genomic positions supported by at least 5 independent exome reads. LLR and Pvalues were calculated on transcriptome and exome data from spinal cord. Decreasing the Fisher Pvalue, and thus the probability observing a genuine RNA editing event is associated with increases in the corresponding LLR scores.
(TIF)

Table S1 List of significant A-to-G substitutions in protein coding regions detected in SRP002274 study aligning short reads onto the complete human genome (hg18 assembly) using Bowtie.
(DOCX)

Table S2 List of significant A-to-G substitutions in protein coding regions detected in SRP002274 study aligning short reads onto the complete human genome (hg18 assembly) using Tophat to take into account the spliced nature of RNA reads.
(DOCX)

Table S3 List of significant A-to-G substitutions in known introns and UTRs detected in transcriptome reads from spinal cord.
(DOCX)
