# Supplementary figures and images for: A Novel Computational Strategy to Identify A-to-I RNA Editing Sites by RNA-Seq Data: De Novo Detection in Human Spinal Cord Tissue
Source: PLoS One. 2012 Sep 5;7(9):e44184. doi: 10.1371/journal.pone.0044184 (PMC3434223; doi:10.1371/journal.pone.0044184)

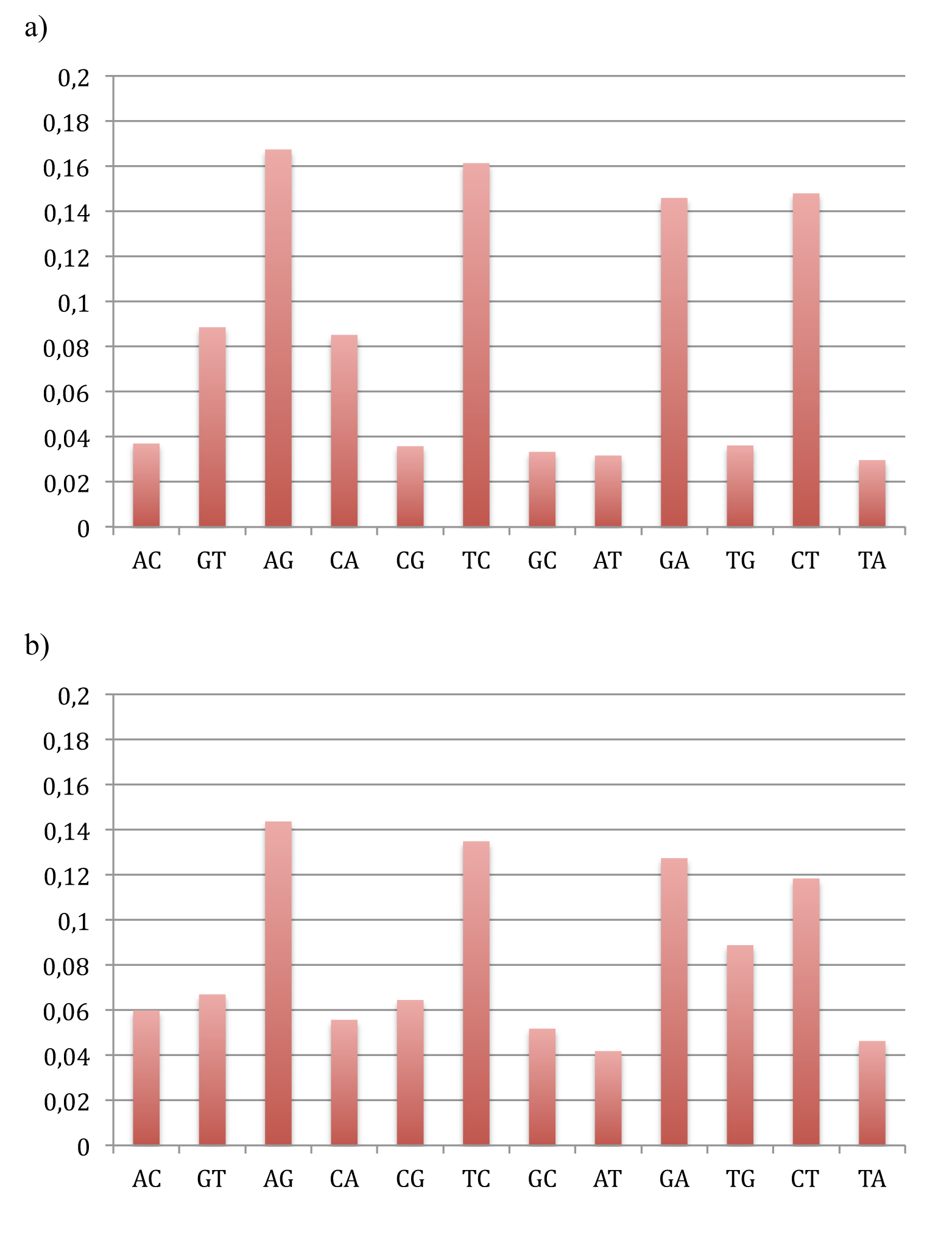

Supplement: Figure S1 — Examples of empirical distribution of base substitutions. Empirical distributions of base substitutions for reads from SRA study SRP002274 (a) using a minimum quality score of 25 and from spinal cord RNA-Seq experiment (b) using a minimum quality score of 30. (TIF) [file pone.0044184.s001.tif]

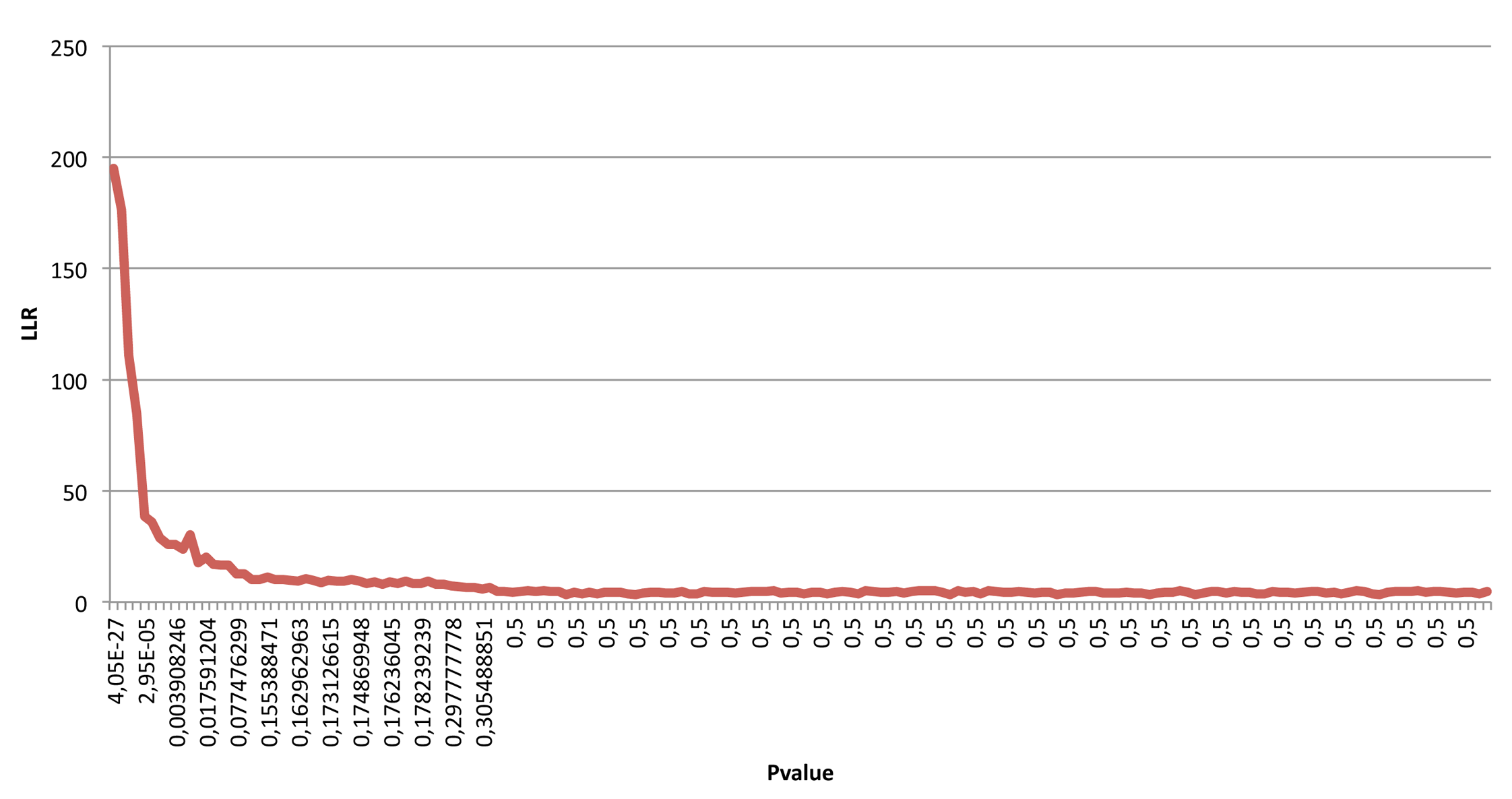

Supplement: Figure S2 — LLR score Vs Fisher Pvalue. Relationship between LLR scores and Fisher Pvalues on 180 genomic positions supported by at least 5 independent exome reads. LLR and Pvalues were calculated on transcriptome and exome data from spinal cord. Decreasing the Fisher Pvalue, and thus the probability observing a genuine RNA editing event is associated with increases in the corresponding LLR scores. (TIF) [file pone.0044184.s002.tif]
