# Supplementary material for: A Novel Computational Strategy to Identify A-to-I RNA Editing Sites by RNA-Seq Data: De Novo Detection in Human Spinal Cord Tissue
Source: PLoS One. 2012 Sep 5;7(9):e44184. doi: 10.1371/journal.pone.0044184 (PMC3434223; doi:10.1371/journal.pone.0044184)
Supplement: Table S1 — List of significant A-to-G substitutions in protein coding regions detected in SRP002274 study aligning short reads onto the complete human genome (hg18 assembly) using Bowtie. (DOCX) [file pone.0044184.s003.docx]

**Supporting Table S1.** List of significant A-to-G substitutions in protein coding regions detected in SRP002274 study aligning short reads onto the complete human genome (hg18 assembly) using Bowtie.

| **Position** | **Gene** | **Ref** | **SubType** | **Loc** | **CovR** | **BaseCountR [A, C, G, T]** | **%Editing** | **Pvalue** | **DARNED** |
| --- | --- | --- | --- | --- | --- | --- | --- | --- | --- |
| chr1:16006501 | UQCRHL | T | TC | CDS | 225 | [0, 223, 0, 2] | 99,11 | 5,21E-128 | 0 |
| chr1:16006499 | UQCRHL | T | TC | CDS | 156 | [0, 155, 0, 1] | 99,36 | 6,46E-89 | 0 |
| chr12:123962441 | UBC | T | TC | CDS | 340 | [0, 189, 0, 151] | 55,59 | 1,31E-71 | 0 |
| chr12:123964037 | UBC | T | TC | CDS | 254 | [0, 149, 0, 105] | 58,66 | 2,79E-57 | 0 |
| chrX:120010117 | GLUD2 | A | AG | CDS | 94 | [0, 0, 94, 0] | 100,00 | 4,17E-54 | 0 |
| chrX:120010787 | GLUD2 | A | AG | CDS | 86 | [1, 0, 85, 0] | 98,84 | 2,03E-47 | 0 |
| chrX:120009899 | GLUD2 | A | AG | CDS | 59 | [0, 0, 59, 0] | 100,00 | 2,46E-33 | 0 |
| chr4:57670991* | IGFBP7 | T | TC | CDS | 161 | [0, 89, 0, 72] | 55,28 | 3,43E-33 | 0 |
| chr13:24569080 | PABPC3 | A | AG | CDS | 48 | [0, 0, 48, 0] | 100,00 | 7,61E-27 | 1 |
| chrX:71296770 | FLJ44635 | A | AG | CDS | 48 | [0, 0, 48, 0] | 100,00 | 7,61E-27 | 1 |
| chr1:116739332 | ATP1A1 | A | AG | CDS | 500 | [419, 0, 81, 0] | 16,20 | 5,47E-25 | 0 |
| chr15:73433139* | NEIL1 | A | AG | CDS | 42 | [0, 0, 42, 0] | 100,00 | 2,56E-23 | 0 |
| chr13:24569143 | PABPC3 | A | AG | CDS | 36 | [1, 0, 35, 0] | 97,22 | 2,93E-18 | 0 |
| chr13:24569574 | PABPC3 | A | AG | CDS | 35 | [1, 0, 34, 0] | 97,14 | 1,09E-17 | 0 |
| chr1:224041237* | SRP9 | A | AG | CDS | 63 | [24, 0, 39, 0] | 61,90 | 8,30E-15 | 0 |
| chr15:73433140* | NEIL1 | A | AG | CDS | 39 | [8, 0, 31, 0] | 79,49 | 3,09E-13 | 0 |
| chr12:123962627 | UBC | T | TC | CDS | 167 | [0, 41, 0, 126] | 24,55 | 6,72E-13 | 0 |
| chr13:24569320 | PABPC3 | A | AG | CDS | 16 | [0, 0, 16, 0] | 100,00 | 2,83E-08 | 1 |
| chr19:59638596 | TTYH1 | A | AG | CDS | 88 | [63, 0, 25, 0] | 28,41 | 6,35E-08 | 1 |
| chrX:120009498 | GLUD2 | A | AG | CDS | 14 | [0, 0, 14, 0] | 100,00 | 3,74E-07 | 0 |
| chr1:12829945 | LOC649330 | T | TC | CDS | 13 | [0, 13, 0, 0] | 100,00 | 1,35E-06 | 0 |
| chrX:120009494 | GLUD2 | A | AG | CDS | 12 | [0, 0, 12, 0] | 100,00 | 4,81E-06 | 0 |
| chr11:62214851 | BSCL2 | T | TC | CDS | 122 | [0, 20, 0, 102] | 16,39 | 4,86E-06 | 0 |
| chr17:35503065 | THRA | T | TC | CDS | 192 | [0, 20, 0, 172] | 10,42 | 6,54E-06 | 0 |
| chr17:7158187 | GPS2 | T | TC | CDS | 27 | [0, 15, 0, 12] | 55,56 | 2,29E-05 | 0 |
| chr4:158500744* | GRIA2 | A | AG | CDS | 21 | [7, 0, 14, 0] | 66,67 | 2,53E-05 | 0 |
| chr21:33845189 | SON | A | AG | CDS | 38 | [23, 0, 15, 0] | 39,47 | 5,63E-05 | 0 |
| chr10:104996050 | LOC729020 | A | AG | CDS | 10 | [0, 0, 10, 0] | 100,00 | 5,95E-05 | 0 |
| chr4:158477325* | GRIA2 | A | AG | CDS | 10 | [0, 0, 10, 0] | 100,00 | 5,95E-05 | 0 |
| chr12:123963215 | UBC | T | TC | CDS | 146 | [0, 16, 0, 130] | 10,96 | 9,35E-05 | 0 |
| chr11:67108595 | GSTP1 | A | AG | CDS | 13 | [2, 0, 11, 0] | 84,62 | 0,00010634 | 1 |
| chr19:8484035 | ZNF414 | T | TC | CDS | 42 | [0, 14, 0, 28] | 33,33 | 0,000156562 | 1 |
| chr17:78039231 | NARF | A | AG | CDS | 20 | [8, 0, 12, 0] | 60,00 | 0,000215813 | 0 |
| chr19:45865738 | NUMBL | T | TC | CDS | 32 | [0, 13, 0, 19] | 40,63 | 0,000242132 | 0 |
| chr6:34208881 | GRM4 | T | TC | CDS | 66 | [0, 14, 0, 52] | 21,21 | 0,000249654 | 0 |
| chr7:100669852 | FIS1 | T | TC | CDS | 102 | [0, 14, 0, 88] | 13,73 | 0,000322236 | 0 |
| chr2:267003 | ACP1 | A | AG | CDS | 29 | [17, 0, 12, 0] | 41,38 | 0,000498433 | 0 |
| chr22:43978451 | C22orf9 | T | TC | CDS | 89 | [0, 13, 0, 76] | 14,61 | 0,000610214 | 0 |
| chr16:1758605 | MAPK8IP3 | A | AG | CDS | 91 | [78, 0, 13, 0] | 14,29 | 0,000616124 | 1 |
| chr14:25987370 | NOVA1 | T | TC | CDS | 31 | [0, 11, 0, 20] | 35,48 | 0,001280447 | 0 |
| chr20:35580986* | BLCAP | T | TC | CDS | 55 | [0, 11, 0, 44] | 20,00 | 0,001995744 | 1 |
| chr1:159354389 | PFDN2 | T | TC | CDS | 58 | [0, 11, 0, 47] | 18,97 | 0,002049703 | 0 |
| chr16:357758 | MRPL28 | T | TC | CDS | 68 | [0, 11, 0, 57] | 16,18 | 0,002199008 | 0 |
| chr5:156669386* | CYFIP2 | A | AG | CDS | 14 | [5, 0, 9, 0] | 64,29 | 0,002212052 | 0 |
| chr19:54848545 | SCAF1 | A | AG | CDS | 78 | [67, 0, 11, 0] | 14,10 | 0,002313642 | 0 |
| chr4:57671043* | IGFBP7 | T | TC | CDS | 83 | [0, 11, 0, 72] | 13,25 | 0,002361487 | 0 |
| chr22:49001293 | TUBGCP6 | T | TC | CDS | 56 | [0, 10, 0, 46] | 17,86 | 0,004083414 | 0 |
| chr7:26198675 | HNRNPA2B1 | T | TC | CDS | 74 | [0, 10, 0, 64] | 13,51 | 0,00448849 | 0 |
| chr2:210543858 | UNC80 | A | AG | CDS | 22 | [13, 0, 9, 0] | 40,91 | 0,004670974 | 0 |
| chr6:44228327 | TMEM63B | A | AG | CDS | 43 | [34, 0, 9, 0] | 20,93 | 0,007389652 | 1 |
| chr20:35580977* | BLCAP | T | TC | CDS | 78 | [0, 9, 0, 69] | 11,54 | 0,008836205 | 0 |
| chr7:45090462 | NACAD | T | TC | CDS | 22 | [0, 8, 0, 14] | 36,36 | 0,010624962 | 0 |
| chr12:119563290 | CABP1 | A | AG | CDS | 41 | [33, 0, 8, 0] | 19,51 | 0,014563355 | 0 |
| chr3:49674600 | BSN | A | AG | CDS | 39 | [32, 0, 7, 0] | 17,95 | 0,028207602 | 0 |
| chr4:78198704* | CCNI | T | TC | CDS | 54 | [0, 7, 0, 47] | 12,96 | 0,030122511 | 0 |

*Positions known as edited by literature

Positions are sorted in ascending Pvalue order. For each site corrected Pvalue (FDR), bases supporting the editing event (BaseCountR), coverage depth (CovR), gene name, chromosome position and potential editing extent are reported. Candidate events stored in DARNED database are flagged with 1 in the last column.
