# Supplementary material for: A Novel Computational Strategy to Identify A-to-I RNA Editing Sites by RNA-Seq Data: De Novo Detection in Human Spinal Cord Tissue
Source: PLoS One. 2012 Sep 5;7(9):e44184. doi: 10.1371/journal.pone.0044184 (PMC3434223; doi:10.1371/journal.pone.0044184)
Supplement: Table S3 — List of significant A-to-G substitutions in known introns and UTRs detected in transcriptome reads from spinal cord. (DOCX) [file pone.0044184.s005.docx]

**Supporting Table S3.** List of significant A-to-G substitutions in known introns and UTRs detected in transcriptome reads from spinal cord.

| **Position** | **Gene** | **Ref** | **SubType** | **Loc** | **CovR** | **BaseCountR [A, C, G, T]** | **CovE** | **BaseCountE [A, C, G, T]** | **%Editing** | **Pvalue** | **Alu** | **DARNED** |
| --- | --- | --- | --- | --- | --- | --- | --- | --- | --- | --- | --- | --- |
| chr16:74065752 | CHST6 | T | TC | 3UTR | 29 | [0 ,14 ,0 ,15] | 0 | [0 ,0 ,0 ,0] | 48,28 | 7,82E-05 | 1 | 0 |
| chr12:97662274 | ANKS1B | T | TC | 3UTR | 16 | [0 ,14 ,0 ,2] | 40 | [0 ,0 ,0 ,40] | 87,5 | 3,42E-06 | 0 | 0 |
| chr1:6204811 | ICMT | T | TC | 3UTR | 28 | [0 ,12 ,0 ,16] | 0 | [0 ,0 ,0 ,0] | 42,86 | 0,000470525 | 1 | 1 |
| chr20:3798391 | MAVS | A | AG | 3UTR | 11 | [2 ,0 ,9 ,0] | 0 | [0 ,0 ,0 ,0] | 81,82 | 0,000952608 | 1 | 0 |
| chr1:218298582 | BPNT1 | T | TC | 3UTR | 13 | [0 ,11 ,0 ,2] | 3 | [0 ,0 ,0 ,3] | 84,62 | 0,00010634 | 1 | 0 |
| chr8:42995291 | HOOK3 | A | AG | 3UTR | 27 | [14 ,0 ,13 ,0] | 0 | [0 ,0 ,0 ,0] | 48,15 | 0,000173056 | 1 | 1 |
| chr2:201737112 | CFLAR | A | AG | 3UTR | 10 | [2 ,0 ,8 ,0] | 3 | [3 ,0 ,0 ,0] | 80 | 0,002738747 | 1 | 0 |
| chr14:52312104 | GNPNAT1 | T | TC | 3UTR | 20 | [0 ,11 ,0 ,9] | 8 | [0 ,0 ,0 ,8] | 55 | 0,000623816 | 1 | 1 |
| chr19:18337428 | PGPEP1 | A | AG | 3UTR | 58 | [49 ,0 ,9 ,0] | 0 | [0 ,0 ,0 ,0] | 15,52 | 0,008211274 | 1 | 0 |
| chr12:49611721 | METTL7A | A | AG | 3UTR | 310 | [279 ,0 ,31 ,0] | 0 | [0 ,0 ,0 ,0] | 10 | 3,66E-09 | 1 | 1 |
| chr11:77457357 | NDUFC2 | T | TC | 3UTR | 43 | [0 ,29 ,0 ,14] | 1 | [0 ,0 ,0 ,1] | 67,44 | 2,65E-11 | 1 | 1 |
| chr5:854216 | ZDHHC11 | T | TC | 3UTR | 50 | [0 ,36 ,0 ,14] | 18 | [0 ,0 ,0 ,18] | 72 | 1,38E-14 | 0 | 0 |
| chr9:27317487 | MOBKL2B | T | TC | 3UTR | 19 | [0 ,13 ,0 ,6] | 0 | [0 ,0 ,0 ,0] | 68,42 | 5,45E-05 | 0 | 0 |
| chr10:15157722 | ACBD7 | T | TC | 3UTR | 181 | [0 ,50 ,0 ,131] | 0 | [0 ,0 ,0 ,0] | 27,62 | 5,20E-16 | 1 | 1 |
| chr22:35214756 | FOXRED2 | T | TC | 3UTR | 10 | [0 ,7 ,0 ,3] | 0 | [0 ,0 ,0 ,0] | 70 | 0,009883306 | 1 | 0 |
| chrX:122874451 | XIAP | A | AG | 3UTR | 12 | [4 ,0 ,8 ,0] | 0 | [0 ,0 ,0 ,0] | 66,67 | 0,004711267 | 1 | 0 |
| chr5:115194199 | ATG12 | T | TC | 3UTR | 73 | [0 ,13 ,0 ,60] | 3 | [0 ,0 ,0 ,3] | 17,81 | 0,000553188 | 1 | 0 |
| chr8:11737640 | CTSB | T | TC | 3UTR | 76 | [0 ,17 ,0 ,59] | 0 | [0 ,0 ,0 ,0] | 22,37 | 2,95E-05 | 1 | 1 |
| chr1:202791169 | MDM4 | A | AG | 3UTR | 13 | [1 ,0 ,12 ,0] | 0 | [0 ,0 ,0 ,0] | 92,31 | 1,63E-05 | 1 | 0 |
| chr5:134264651 | TXNDC15 | A | AG | 3UTR | 41 | [12 ,0 ,29 ,0] | 1 | [1 ,0 ,0 ,0] | 70,73 | 1,47E-11 | 1 | 1 |
| chr8:95874214 | DPY19L4 | A | AG | 3UTR | 14 | [0 ,0 ,14 ,0] | 0 | [0 ,0 ,0 ,0] | 100 | 3,74E-07 | 1 | 1 |
| chr10:15157935 | ACBD7 | T | TC | 3UTR | 57 | [0 ,15 ,0 ,42] | 0 | [0 ,0 ,0 ,0] | 26,32 | 0,000101986 | 1 | 0 |
| chr17:46396865 | SPAG9 | T | TC | 3UTR | 14 | [0 ,8 ,0 ,6] | 0 | [0 ,0 ,0 ,0] | 57,14 | 0,006376812 | 1 | 0 |
| chr3:49372327 | RHOA | T | TC | 3UTR | 693 | [0 ,149 ,0 ,544] | 6 | [0 ,0 ,0 ,6] | 21,5 | 1,56E-47 | 0 | 0 |
| chr8:42995875 | HOOK3 | A | AG | 3UTR | 21 | [5 ,0 ,16 ,0] | 0 | [0 ,0 ,0 ,0] | 76,19 | 1,70E-06 | 1 | 0 |
| chr8:102278525 | ZNF706 | T | TC | 3UTR | 17 | [0 ,12 ,0 ,5] | 18 | [0 ,0 ,0 ,18] | 70,59 | 0,000115924 | 0 | 0 |
| chr8:42998008 | HOOK3 | A | AG | 3UTR | 14 | [4 ,0 ,10 ,0] | 2 | [2 ,0 ,0 ,0] | 71,43 | 0,000669548 | 1 | 0 |
| chr17:22664083 | WSB1 | A | AG | 3UTR | 138 | [123 ,0 ,15 ,0] | 0 | [0 ,0 ,0 ,0] | 10,87 | 0,000182382 | 1 | 0 |
| chr20:31756517 | PXMP4 | T | TC | 3UTR | 13 | [0 ,8 ,0 ,5] | 0 | [0 ,0 ,0 ,0] | 61,54 | 0,005583524 | 1 | 0 |
| chr3:40553835 | ZNF621 | A | AG | 3UTR | 21 | [13 ,0 ,8 ,0] | 0 | [0 ,0 ,0 ,0] | 38,1 | 0,010242888 | 1 | 0 |
| chr5:150628186 | GM2A | A | AG | 3UTR | 109 | [96 ,0 ,13 ,0] | 0 | [0 ,0 ,0 ,0] | 11,93 | 0,00066064 | 1 | 0 |
| chr15:71382392 | NEO1 | A | AG | 3UTR | 27 | [18 ,0 ,9 ,0] | 3 | [3 ,0 ,0 ,0] | 33,33 | 0,005640474 | 0 | 0 |
| chr14:74272165 | FCF1 | A | AG | 3UTR | 24 | [9 ,0 ,15 ,0] | 3 | [3 ,0 ,0 ,0] | 62,5 | 1,42E-05 | 1 | 1 |
| chr3:49436827 | NICN1 | T | TC | 3UTR | 13 | [0 ,8 ,0 ,5] | 5 | [0 ,0 ,0 ,5] | 61,54 | 0,005583524 | 0 | 1 |
| chr5:134264180 | TXNDC15 | A | AG | 3UTR | 40 | [20 ,0 ,20 ,0] | 2 | [2 ,0 ,0 ,0] | 50 | 5,59E-07 | 1 | 0 |
| chr12:67523340 | MDM2 | A | AG | 3UTR | 60 | [47 ,0 ,13 ,0] | 8 | [8 ,0 ,0 ,0] | 21,67 | 0,000489022 | 0 | 0 |
| chrX:122874846 | XIAP | A | AG | 3UTR | 51 | [34 ,0 ,17 ,0] | 0 | [0 ,0 ,0 ,0] | 33,33 | 1,72E-05 | 1 | 1 |
| chr1:202791178 | MDM4 | A | AG | 3UTR | 15 | [4 ,0 ,11 ,0] | 0 | [0 ,0 ,0 ,0] | 73,33 | 0,000241984 | 1 | 0 |
| chr1:200820860 | PPP1R12B | A | AG | 3UTR | 57 | [46 ,0 ,11 ,0] | 0 | [0 ,0 ,0 ,0] | 19,3 | 0,002032264 | 1 | 1 |
| chr3:43365261 | SNRK | A | AG | 3UTR | 31 | [18 ,0 ,13 ,0] | 18 | [18 ,0 ,0 ,0] | 41,94 | 0,000228998 | 0 | 0 |
| chr6:169847478 | C6orf120 | T | TC | 3UTR | 27 | [0 ,11 ,0 ,16] | 27 | [0 ,0 ,0 ,27] | 40,74 | 0,001076967 | 0 | 0 |
| chr1:38100165 | INPP5B | T | TC | 3UTR | 10 | [0 ,9 ,0 ,1] | 0 | [0 ,0 ,0 ,0] | 90 | 0,000546667 | 1 | 1 |
| chr8:26324144 | BNIP3L | A | AG | 3UTR | 74 | [55 ,0 ,19 ,0] | 13 | [13 ,0 ,0 ,0] | 25,68 | 6,03E-06 | 0 | 0 |
| chr11:8664416 | RPL27A | A | AG | 3UTR | 17 | [8 ,0 ,9 ,0] | 0 | [0 ,0 ,0 ,0] | 52,94 | 0,003299963 | 1 | 0 |
| chr17:46396691 | SPAG9 | T | TC | 3UTR | 237 | [0 ,84 ,0 ,153] | 4 | [0 ,0 ,0 ,4] | 35,44 | 3,33E-28 | 1 | 0 |
| chr19:18338420 | PGPEP1 | A | AG | 3UTR | 31 | [22 ,0 ,9 ,0] | 0 | [0 ,0 ,0 ,0] | 29,03 | 0,006225085 | 1 | 0 |
| chrX:122874121 | XIAP | A | AG | 3UTR | 25 | [14 ,0 ,11 ,0] | 0 | [0 ,0 ,0 ,0] | 44 | 0,000960755 | 1 | 0 |
| chr4:57021090 | PAICS | A | AG | 3UTR | 25 | [15 ,0 ,10 ,0] | 0 | [0 ,0 ,0 ,0] | 40 | 0,002307035 | 1 | 0 |
| chr19:42721329 | ZNF793 | A | AG | 3UTR | 15 | [6 ,0 ,9 ,0] | 0 | [0 ,0 ,0 ,0] | 60 | 0,002598701 | 1 | 0 |
| chr5:67632579 | PIK3R1 | A | AG | 3UTR | 75 | [66 ,0 ,9 ,0] | 46 | [46 ,0 ,0 ,0] | 12 | 0,008762828 | 0 | 0 |
| chrX:122874323 | XIAP | A | AG | 3UTR | 15 | [4 ,0 ,11 ,0] | 0 | [0 ,0 ,0 ,0] | 73,33 | 0,000241984 | 1 | 0 |
| chr13:36318237 | SMAD9 | T | TC | 3UTR | 21 | [0 ,19 ,0 ,2] | 0 | [0 ,0 ,0 ,0] | 90,48 | 8,62E-09 | 1 | 0 |
| chr12:49610703 | METTL7A | A | AG | 3UTR | 33 | [21 ,0 ,12 ,0] | 0 | [0 ,0 ,0 ,0] | 36,36 | 0,000600626 | 1 | 1 |
| chr19:13745096 | MRI1 | A | AG | 3UTR | 32 | [21 ,0 ,11 ,0] | 1 | [1 ,0 ,0 ,0] | 34,38 | 0,001325911 | 1 | 1 |
| chr5:126408402 | FLJ44606 | T | TC | 3UTR | 83 | [0 ,81 ,0 ,2] | 8 | [0 ,0 ,0 ,8] | 97,59 | 4,94E-44 | 1 | 0 |
| chr12:49610906 | METTL7A | A | AG | 3UTR | 60 | [35 ,0 ,25 ,0] | 0 | [0 ,0 ,0 ,0] | 41,67 | 2,09E-08 | 1 | 1 |
| chr20:34954391 | SAMHD1 | T | TC | 3UTR | 59 | [0 ,10 ,0 ,49] | 2 | [0 ,0 ,0 ,2] | 16,95 | 0,004166614 | 1 | 0 |
| chr1:154119685 | SYT11 | A | AG | 3UTR | 52 | [42 ,0 ,10 ,0] | 1 | [1 ,0 ,0 ,0] | 19,23 | 0,003959048 | 1 | 0 |
| chr8:11738899 | CTSB | T | TC | 3UTR | 20 | [0 ,17 ,0 ,3] | 5 | [0 ,0 ,0 ,5] | 85 | 2,03E-07 | 1 | 1 |
| chr10:74678737 | MRPS16 | T | TC | 3UTR | 50 | [0 ,15 ,0 ,35] | 0 | [0 ,0 ,0 ,0] | 30 | 8,73E-05 | 1 | 0 |
| chr12:49610469 | METTL7A | A | AG | 3UTR | 26 | [17 ,0 ,9 ,0] | 0 | [0 ,0 ,0 ,0] | 34,62 | 0,005470917 | 1 | 1 |
| chr10:15157799 | ACBD7 | T | TC | 3UTR | 70 | [0 ,15 ,0 ,55] | 0 | [0 ,0 ,0 ,0] | 21,43 | 0,000124028 | 1 | 1 |
| chr12:63394777 | GNS | T | TC | 3UTR | 83 | [0 ,12 ,0 ,71] | 18 | [0 ,0 ,0 ,18] | 14,46 | 0,001188841 | 0 | 0 |
| chr1:153196927 | PYGO2 | T | TC | 3UTR | 31 | [0 ,28 ,0 ,3] | 18 | [0 ,0 ,0 ,18] | 90,32 | 3,41E-13 | 0 | 0 |
| chr8:11738894 | CTSB | T | TC | 3UTR | 39 | [0 ,12 ,0 ,27] | 5 | [0 ,0 ,0 ,5] | 30,77 | 0,000728556 | 1 | 0 |
| chr15:43563105 | SLC30A4 | T | TC | 3UTR | 33 | [0 ,12 ,0 ,21] | 7 | [0 ,0 ,0 ,7] | 36,36 | 0,000600626 | 1 | 0 |
| chr8:131133864 | ASAP1 | T | TC | 3UTR | 19 | [0 ,9 ,0 ,10] | 35 | [0 ,0 ,0 ,35] | 47,37 | 0,003908246 | 0 | 0 |
| chr19:18338923 | PGPEP1 | A | AG | 3UTR | 53 | [39 ,0 ,14 ,0] | 0 | [0 ,0 ,0 ,0] | 26,42 | 0,000206154 | 1 | 0 |
| chr14:20998691 | RAB2B | T | TC | 3UTR | 11 | [0 ,9 ,0 ,2] | 0 | [0 ,0 ,0 ,0] | 81,82 | 0,000952608 | 1 | 0 |
| chr17:76705948 | AATK | T | TC | 3UTR | 14 | [0 ,14 ,0 ,0] | 2 | [0 ,0 ,0 ,2] | 100 | 3,74E-07 | 0 | 0 |
| chr3:157741982 | SSR3 | T | TC | 3UTR | 63 | [0 ,14 ,0 ,49] | 1 | [0 ,0 ,0 ,1] | 22,22 | 0,000240776 | 1 | 0 |
| chr10:15157716 | ACBD7 | T | TC | 3UTR | 143 | [0 ,85 ,0 ,58] | 0 | [0 ,0 ,0 ,0] | 59,44 | 1,84E-32 | 1 | 1 |
| chr19:40462014 | USF2 | A | AG | 3UTR | 49 | [27 ,0 ,22 ,0] | 0 | [0 ,0 ,0 ,0] | 44,9 | 1,70E-07 | 0 | 0 |
| chr19:13745611 | MRI1 | A | AG | 3UTR | 40 | [17 ,0 ,23 ,0] | 0 | [0 ,0 ,0 ,0] | 57,5 | 2,23E-08 | 1 | 1 |
| chr17:26885675 | RAB11FIP4 | A | AG | 3UTR | 57 | [47 ,0 ,10 ,0] | 4 | [4 ,0 ,0 ,0] | 17,54 | 0,004112031 | 1 | 0 |
| chr8:11737995 | CTSB | T | TC | 3UTR | 110 | [0 ,25 ,0 ,85] | 0 | [0 ,0 ,0 ,0] | 22,73 | 9,66E-08 | 1 | 0 |
| chr19:21266322 | ZNF708 | T | TC | 3UTR | 15 | [0 ,13 ,0 ,2] | 0 | [0 ,0 ,0 ,0] | 86,67 | 1,09E-05 | 1 | 0 |
| chr1:89421967 | GBP4 | T | TC | 3UTR | 35 | [0 ,29 ,0 ,6] | 0 | [0 ,0 ,0 ,0] | 82,86 | 1,03E-12 | 1 | 1 |
| chr15:36436623 | SPRED1 | A | AG | 3UTR | 45 | [36 ,0 ,9 ,0] | 47 | [47 ,0 ,0 ,0] | 20 | 0,007528439 | 0 | 0 |
| chr14:74272757 | FCF1 | A | AG | 3UTR | 27 | [7 ,0 ,20 ,0] | 0 | [0 ,0 ,0 ,0] | 74,07 | 4,66E-08 | 1 | 1 |
| chr7:65256801 | CRCP | A | AG | 3UTR | 29 | [19 ,0 ,10 ,0] | 0 | [0 ,0 ,0 ,0] | 34,48 | 0,002703067 | 1 | 0 |
| chr1:6204911 | ICMT | T | TC | 3UTR | 18 | [0 ,11 ,0 ,7] | 0 | [0 ,0 ,0 ,0] | 61,11 | 0,000472483 | 1 | 1 |
| chr17:71451782 | ACOX1 | T | TC | 3UTR | 11 | [0 ,9 ,0 ,2] | 0 | [0 ,0 ,0 ,0] | 81,82 | 0,000952608 | 1 | 0 |
| chr22:34992152 | APOL1 | A | AG | 3UTR | 15 | [5 ,0 ,10 ,0] | 1 | [1 ,0 ,0 ,0] | 66,67 | 0,000849575 | 1 | 0 |
| chr10:101982800 | CWF19L1 | T | TC | 3UTR | 31 | [0 ,25 ,0 ,6] | 0 | [0 ,0 ,0 ,0] | 80,65 | 1,10E-10 | 1 | 1 |
| chr13:19144359 | MPHOSPH8 | A | AG | 3UTR | 28 | [16 ,0 ,12 ,0] | 1 | [1 ,0 ,0 ,0] | 42,86 | 0,000470525 | 1 | 1 |
| chr10:80816070 | ZCCHC24 | T | TC | 3UTR | 51 | [0 ,13 ,0 ,38] | 1 | [0 ,0 ,0 ,1] | 25,49 | 0,00043025 | 0 | 0 |
| chr16:74065314 | CHST6 | T | TC | 3UTR | 47 | [0 ,11 ,0 ,36] | 0 | [0 ,0 ,0 ,0] | 23,4 | 0,00182362 | 1 | 0 |
| chr3:151828141 | SELT | A | AG | 3UTR | 142 | [117 ,0 ,25 ,0] | 10 | [10 ,0 ,0 ,0] | 17,61 | 1,38E-07 | 0 | 0 |
| chr8:95873926 | DPY19L4 | A | AG | 3UTR | 20 | [0 ,0 ,20 ,0] | 0 | [0 ,0 ,0 ,0] | 100 | 1,52E-10 | 1 | 1 |
| chr17:46396731 | SPAG9 | T | TC | 3UTR | 177 | [0 ,34 ,0 ,143] | 0 | [0 ,0 ,0 ,0] | 19,21 | 2,00E-10 | 0 | 0 |
| chr11:758383 | PDDC1 | T | TC | 3UTR | 24 | [0 ,10 ,0 ,14] | 0 | [0 ,0 ,0 ,0] | 41,67 | 0,002193664 | 1 | 0 |
| chr10:15159232 | ACBD7 | T | TC | 3UTR | 23 | [0 ,17 ,0 ,6] | 0 | [0 ,0 ,0 ,0] | 73,91 | 8,36E-07 | 1 | 0 |
| chr5:137919342 | HSPA9 | T | TC | 3UTR | 100 | [0 ,72 ,0 ,28] | 16 | [0 ,11 ,0 ,5] | 72 | 8,57E-30 | 0 | 0 |
| chr10:15159230 | ACBD7 | T | TC | 3UTR | 21 | [0 ,12 ,0 ,9] | 0 | [0 ,0 ,0 ,0] | 57,14 | 0,000249856 | 0 | 0 |
| chr19:21266252 | ZNF708 | T | TC | 3UTR | 66 | [0 ,12 ,0 ,54] | 10 | [0 ,0 ,0 ,10] | 18,18 | 0,001072075 | 0 | 0 |
| chr18:27904939 | RNF125 | A | AG | 3UTR | 69 | [59 ,0 ,10 ,0] | 0 | [0 ,0 ,0 ,0] | 14,49 | 0,004395615 | 1 | 0 |
| chr8:6603969 | AGPAT5 | A | AG | 3UTR | 63 | [41 ,0 ,22 ,0] | 18 | [18 ,0 ,0 ,0] | 34,92 | 3,69E-07 | 1 | 0 |
| chr12:67523277 | MDM2 | A | AG | 3UTR | 15 | [7 ,0 ,8 ,0] | 13 | [13 ,0 ,0 ,0] | 53,33 | 0,007096452 | 0 | 0 |
| chr1:159233058 | F11R | T | TC | 3UTR | 10 | [0 ,9 ,0 ,1] | 0 | [0 ,0 ,0 ,0] | 90 | 0,000546667 | 0 | 1 |
| chr14:69903808 | SYNJ2BP | T | TC | 3UTR | 67 | [0 ,9 ,0 ,58] | 1 | [0 ,0 ,0 ,1] | 13,43 | 0,008536499 | 1 | 0 |
| chr17:46396763 | SPAG9 | T | TC | 3UTR | 45 | [0 ,31 ,0 ,14] | 0 | [0 ,0 ,0 ,0] | 68,89 | 3,16E-12 | 1 | 0 |
| chr17:41461232 | MAPT | A | AG | 3UTR | 165 | [70 ,0 ,95 ,0] | 3 | [3 ,0 ,0 ,0] | 57,58 | 5,78E-36 | 1 | 0 |
| chr3:180598105 | GNB4 | T | TC | 3UTR | 40 | [0 ,18 ,0 ,22] | 26 | [0 ,0 ,0 ,26] | 45 | 4,03E-06 | 0 | 0 |
| chr21:33651978 | IFNAR1 | A | AG | 3UTR | 33 | [14 ,0 ,19 ,0] | 2 | [2 ,0 ,0 ,0] | 57,58 | 6,79E-07 | 0 | 0 |
| chr17:46396826 | SPAG9 | T | TC | 3UTR | 59 | [0 ,17 ,0 ,42] | 0 | [0 ,0 ,0 ,0] | 28,81 | 2,17E-05 | 1 | 0 |
| chr1:28698777 | PHACTR4 | A | AG | 3UTR | 14 | [1 ,0 ,13 ,0] | 0 | [0 ,0 ,0 ,0] | 92,86 | 4,91E-06 | 1 | 1 |
| chr10:15159238 | ACBD7 | T | TC | 3UTR | 21 | [0 ,10 ,0 ,11] | 0 | [0 ,0 ,0 ,0] | 47,62 | 0,001812788 | 1 | 0 |
| chr11:758844 | PDDC1 | T | TC | 3UTR | 32 | [0 ,17 ,0 ,15] | 0 | [0 ,0 ,0 ,0] | 53,12 | 5,16E-06 | 0 | 0 |
| chr10:101982773 | CWF19L1 | T | TC | 3UTR | 17 | [0 ,11 ,0 ,6] | 0 | [0 ,0 ,0 ,0] | 64,71 | 0,000394964 | 1 | 1 |
| chr1:92747755 | EVI5 | T | TC | 3UTR | 26 | [0 ,10 ,0 ,16] | 2 | [0 ,0 ,0 ,2] | 38,46 | 0,002414276 | 1 | 0 |
| chr17:72286550 | MFSD11 | A | AG | 3UTR | 12 | [4 ,0 ,8 ,0] | 0 | [0 ,0 ,0 ,0] | 66,67 | 0,004711267 | 1 | 1 |
| chrX:122874729 | XIAP | A | AG | 3UTR | 44 | [25 ,0 ,19 ,0] | 0 | [0 ,0 ,0 ,0] | 43,18 | 2,07E-06 | 1 | 1 |
| chr5:108698713 | PJA2 | T | TC | 3UTR | 42 | [0 ,32 ,0 ,10] | 41 | [0 ,0 ,0 ,41] | 76,19 | 2,53E-13 | 1 | 0 |
| chr17:23751849 | SARM1 | A | AG | 3UTR | 17 | [0 ,0 ,17 ,0] | 0 | [0 ,0 ,0 ,0] | 100 | 7,71E-09 | 0 | 0 |
| chr20:3798213 | MAVS | A | AG | 3UTR | 17 | [7 ,0 ,10 ,0] | 0 | [0 ,0 ,0 ,0] | 58,82 | 0,001198863 | 0 | 0 |
| chr12:49610773 | METTL7A | A | AG | 3UTR | 49 | [25 ,0 ,24 ,0] | 0 | [0 ,0 ,0 ,0] | 48,98 | 2,32E-08 | 1 | 1 |
| chr6:52976042 | ICK | T | TC | 3UTR | 40 | [0 ,9 ,0 ,31] | 23 | [0 ,0 ,0 ,23] | 22,5 | 0,007157772 | 1 | 0 |
| chr1:3720260 | KIAA0562 | T | TC | 3UTR | 25 | [0 ,23 ,0 ,2] | 1 | [0 ,0 ,0 ,1] | 92 | 6,19E-11 | 0 | 0 |
| chr15:49485014 | GLDN | A | AG | 3UTR | 70 | [48 ,0 ,22 ,0] | 4 | [4 ,0 ,0 ,0] | 31,43 | 4,72E-07 | 1 | 0 |
| chr8:1717804 | CLN8 | A | AG | 3UTR | 41 | [30 ,0 ,11 ,0] | 0 | [0 ,0 ,0 ,0] | 26,83 | 0,001658891 | 1 | 0 |
| chr8:11737935 | CTSB | T | TC | 3UTR | 62 | [0 ,11 ,0 ,51] | 0 | [0 ,0 ,0 ,0] | 17,74 | 0,002114517 | 1 | 1 |
| chr15:62221495 | SNX1 | A | AG | 3UTR | 11 | [3 ,0 ,8 ,0] | 0 | [0 ,0 ,0 ,0] | 72,73 | 0,003759398 | 1 | 0 |
| chr1:78115317 | FAM73A | A | AG | 3UTR | 20 | [10 ,0 ,10 ,0] | 10 | [10 ,0 ,0 ,0] | 50 | 0,001671026 | 1 | 0 |
| chrX:118556553 | CXorf56 | T | TC | 3UTR | 15 | [0 ,8 ,0 ,7] | 0 | [0 ,0 ,0 ,0] | 53,33 | 0,007096452 | 1 | 1 |
| chr7:38730867 | VPS41 | T | TC | 3UTR | 10 | [0 ,7 ,0 ,3] | 4 | [0 ,0 ,0 ,4] | 70 | 0,009883306 | 1 | 1 |
| chr9:32446380 | DDX58 | T | TC | 3UTR | 11 | [0 ,8 ,0 ,3] | 6 | [0 ,0 ,0 ,6] | 72,73 | 0,003759398 | 1 | 1 |
| chr8:42995334 | HOOK3 | A | AG | 3UTR | 42 | [30 ,0 ,12 ,0] | 0 | [0 ,0 ,0 ,0] | 28,57 | 0,000782925 | 0 | 1 |
| chr6:52468378 | EFHC1 | A | AG | 3UTR | 13 | [1 ,0 ,12 ,0] | 0 | [0 ,0 ,0 ,0] | 92,31 | 1,63E-05 | 1 | 1 |
| chr22:35214775 | FOXRED2 | T | TC | 3UTR | 10 | [0 ,10 ,0 ,0] | 0 | [0 ,0 ,0 ,0] | 100 | 5,95E-05 | 1 | 1 |
| chr2:203878442 | CYP20A1 | A | AG | 3UTR | 27 | [17 ,0 ,10 ,0] | 5 | [5 ,0 ,0 ,0] | 37,04 | 0,002515779 | 1 | 1 |
| chr19:44051069 | RINL | T | TC | 3UTR | 12 | [0 ,10 ,0 ,2] | 1 | [0 ,0 ,0 ,1] | 83,33 | 0,000322097 | 1 | 0 |
| chr1:218297886 | BPNT1 | T | TC | 3UTR | 102 | [0 ,69 ,0 ,33] | 2 | [0 ,0 ,0 ,2] | 67,65 | 1,19E-27 | 1 | 0 |
| chr3:10167608 | VHL | A | AG | 3UTR | 12 | [3 ,0 ,9 ,0] | 0 | [0 ,0 ,0 ,0] | 75 | 0,001379728 | 1 | 1 |
| chr11:759738 | PDDC1 | T | TC | 3UTR | 20 | [0 ,9 ,0 ,11] | 0 | [0 ,0 ,0 ,0] | 45 | 0,004180867 | 1 | 1 |
| chr4:55931746 | SRD5A3 | A | AG | 3UTR | 12 | [0 ,0 ,12 ,0] | 3 | [3 ,0 ,0 ,0] | 100 | 4,81E-06 | 1 | 0 |
| chr11:757979 | PDDC1 | T | TC | 3UTR | 24 | [0 ,12 ,0 ,12] | 0 | [0 ,0 ,0 ,0] | 50 | 0,000349331 | 1 | 1 |
| chr4:57021632 | PAICS | A | AG | 3UTR | 19 | [9 ,0 ,10 ,0] | 8 | [8 ,0 ,0 ,0] | 52,63 | 0,001521424 | 1 | 0 |
| chr5:79959186 | DHFR | T | TC | 3UTR | 15 | [0 ,9 ,0 ,6] | 0 | [0 ,0 ,0 ,0] | 60 | 0,002598701 | 1 | 1 |
| chr2:128667577 | UGGT1 | A | AG | 3UTR | 26 | [14 ,0 ,12 ,0] | 2 | [2 ,0 ,0 ,0] | 46,15 | 0,000411803 | 1 | 0 |
| chr16:74066255 | CHST6 | T | TC | 3UTR | 33 | [0 ,10 ,0 ,23] | 0 | [0 ,0 ,0 ,0] | 30,3 | 0,003024023 | 1 | 0 |
| chr4:57021636 | PAICS | A | AG | 3UTR | 23 | [7 ,0 ,16 ,0] | 7 | [7 ,0 ,0 ,0] | 69,57 | 3,28E-06 | 1 | 0 |
| chr19:13745525 | MRI1 | A | AG | 3UTR | 20 | [0 ,0 ,20 ,0] | 0 | [0 ,0 ,0 ,0] | 100 | 1,52E-10 | 1 | 1 |
| chr3:180598840 | GNB4 | T | TC | 3UTR | 13 | [0 ,8 ,0 ,5] | 2 | [0 ,0 ,0 ,2] | 61,54 | 0,005583524 | 1 | 0 |
| chr12:67523801 | MDM2 | A | AG | 3UTR | 28 | [6 ,0 ,22 ,0] | 16 | [16 ,0 ,0 ,0] | 78,57 | 3,36E-09 | 1 | 0 |
| chr13:19144402 | MPHOSPH8 | A | AG | 3UTR | 46 | [35 ,0 ,11 ,0] | 1 | [1 ,0 ,0 ,0] | 23,91 | 0,001798614 | 0 | 1 |
| chr16:10532609 | EMP2 | T | TC | 3UTR | 76 | [0 ,9 ,0 ,67] | 0 | [0 ,0 ,0 ,0] | 11,84 | 0,008787905 | 1 | 0 |
| chr10:72309402 | SGPL1 | A | AG | 3UTR | 118 | [93 ,0 ,25 ,0] | 4 | [4 ,0 ,0 ,0] | 21,19 | 1,08E-07 | 1 | 0 |
| chr5:79959067 | DHFR | T | TC | 3UTR | 24 | [0 ,15 ,0 ,9] | 0 | [0 ,0 ,0 ,0] | 62,5 | 1,42E-05 | 0 | 0 |
| chr17:46396768 | SPAG9 | T | TC | 3UTR | 35 | [0 ,26 ,0 ,9] | 0 | [0 ,0 ,0 ,0] | 74,29 | 1,37E-10 | 1 | 0 |
| chr10:15157835 | ACBD7 | T | TC | 3UTR | 22 | [0 ,9 ,0 ,13] | 0 | [0 ,0 ,0 ,0] | 40,91 | 0,004670974 | 1 | 1 |
| chr19:13744962 | MRI1 | A | AG | 3UTR | 17 | [9 ,0 ,8 ,0] | 0 | [0 ,0 ,0 ,0] | 47,06 | 0,008342603 | 1 | 0 |
| chr1:19459088 | MRTO4 | A | AG | 3UTR | 17 | [6 ,0 ,11 ,0] | 1 | [1 ,0 ,0 ,0] | 64,71 | 0,000394964 | 1 | 0 |
| chr5:134264629 | TXNDC15 | A | AG | 3UTR | 11 | [2 ,0 ,9 ,0] | 1 | [1 ,0 ,0 ,0] | 81,82 | 0,000952608 | 1 | 1 |
| chr6:169847478 | PHF10 | T | TC | Intron | 27 | [0 ,11 ,0 ,16] | 27 | [0 ,0 ,0 ,27] | 40,74 | 0,001076967 | 1 | 0 |
| chr12:97662274 | ANKS1B | T | TC | Intron | 16 | [0 ,14 ,0 ,2] | 40 | [0 ,0 ,0 ,40] | 87,5 | 3,42E-06 | 0 | 0 |
| chr20:34954391 | SAMHD1 | T | TC | Intron | 59 | [0 ,10 ,0 ,49] | 2 | [0 ,0 ,0 ,2] | 16,95 | 0,004166614 | 1 | 0 |
| chr3:143386252 | GK5 | T | TC | Intron | 14 | [0 ,14 ,0 ,0] | 0 | [0 ,0 ,0 ,0] | 100 | 3,74E-07 | 1 | 1 |
| chr7:99648429 | GATS | T | TC | Intron | 31 | [0 ,23 ,0 ,8] | 0 | [0 ,0 ,0 ,0] | 74,19 | 2,55E-09 | 1 | 1 |
| chr19:17726476 | FCHO1 | A | AG | Intron | 12 | [0 ,0 ,12 ,0] | 0 | [0 ,0 ,0 ,0] | 100 | 4,81E-06 | 0 | 0 |
| chr2:130836287 | PTPN18 | A | AG | Intron | 10 | [0 ,0 ,10 ,0] | 0 | [0 ,0 ,0 ,0] | 100 | 5,95E-05 | 1 | 0 |
| chr5:134291094 | PCBD2 | A | AG | Intron | 11 | [2 ,0 ,9 ,0] | 0 | [0 ,0 ,0 ,0] | 81,82 | 0,000952608 | 0 | 0 |
| chr1:206010008 | CD46 | A | AG | Intron | 57 | [36 ,0 ,21 ,0] | 0 | [0 ,0 ,0 ,0] | 36,84 | 6,87E-07 | 1 | 0 |
| chr20:55367612 | RAE1 | A | AG | Intron | 20 | [0 ,0 ,20 ,0] | 0 | [0 ,0 ,0 ,0] | 100 | 1,52E-10 | 0 | 0 |
| chr1:206009908 | CD46 | A | AG | Intron | 13 | [4 ,0 ,9 ,0] | 0 | [0 ,0 ,0 ,0] | 69,23 | 0,001803742 | 1 | 1 |
| chr9:130229822 | CERCAM | A | AG | Intron | 19 | [6 ,0 ,13 ,0] | 0 | [0 ,0 ,0 ,0] | 68,42 | 5,45E-05 | 1 | 0 |
| chr5:134291188 | PCBD2 | A | AG | Intron | 247 | [0 ,0 ,247 ,0] | 1 | [0 ,0 ,1 ,0] | 100 | 1,35E-145 | 0 | 0 |
| chr5:134291288 | PCBD2 | A | AG | Intron | 221 | [13 ,0 ,208 ,0] | 4 | [0 ,0 ,4 ,0] | 94,12 | 3,31E-109 | 0 | 0 |
| chr5:134290528 | PCBD2 | A | AG | Intron | 20 | [0 ,0 ,20 ,0] | 0 | [0 ,0 ,0 ,0] | 100 | 1,52E-10 | 0 | 0 |
| chr5:134291611 | PCBD2 | A | AG | Intron | 149 | [0 ,0 ,149 ,0] | 0 | [0 ,0 ,0 ,0] | 100 | 6,38E-87 | 0 | 0 |
| chr1:229765834 | TSNAX | A | AG | Intron | 24 | [8 ,0 ,16 ,0] | 0 | [0 ,0 ,0 ,0] | 66,67 | 4,24E-06 | 0 | 0 |
| chr5:134290270 | PCBD2 | A | AG | Intron | 13 | [1 ,0 ,12 ,0] | 0 | [0 ,0 ,0 ,0] | 92,31 | 1,63E-05 | 0 | 0 |
| chr6:13693217 | SIRT5 | A | AG | Intron | 10 | [0 ,0 ,10 ,0] | 0 | [0 ,0 ,0 ,0] | 100 | 5,95E-05 | 0 | 0 |
| chr5:134291101 | PCBD2 | A | AG | Intron | 16 | [0 ,0 ,16 ,0] | 0 | [0 ,0 ,0 ,0] | 100 | 2,83E-08 | 0 | 0 |
| chr1:224041237 | SRP9 | A | AG | Intron | 30 | [19 ,0 ,11 ,0] | 0 | [0 ,0 ,0 ,0] | 36,67 | 0,001232931 | 1 | 0 |
| chr1:143960195 | NOTCH2NL | A | AG | 5UTR | 91 | [75 ,0 ,16 ,0] | 73 | [73 ,0 ,0 ,0] | 17.58 | 7,24E-05 | 0 | 0 |
| chr19:17726476 | FCHO1 | A | AG | 5UTR | 12 | [0 ,0 ,12 ,0] | 0 | [0 ,0 ,0 ,0] | 100.00.00 | 4,81E-06 | 0 | 0 |

Exome support is also reported for each position as well as Pvalue. BaseCountR reports the distribution of supported RNA-Seq bases, whereas BaseCountE indicates the distribution of supported exome bases. CovR and CovE are the coverage per RNA-Seq and exome, respectively. Positions falling in Alu elements are flagged with 1 in the Alu column. Potential editing sites stored also in DARNED database are flagged with 1 in the DARNED column.
